# Niosome as a promising tool for increasing the effectiveness of anti-inflammatory compounds

**DOI:** 10.17179/excli2023-6868

**Published:** 2024-02-07

**Authors:** Mohammad Saleh Fadaei, Mohammad Reza Fadaei, Amir Emad Kheirieh, Pouria Rahmanian-Devin, Mohammad Mahdi Dabbaghi, Kiarash Nazari Tavallaei, Abouzar Shafaghi, Hooman Hatami, Vafa Baradaran Rahimi, Ali Nokhodchi, Vahid Reza Askari

**Affiliations:** 1 Applied Biomedical Research Center, Mashhad University of Medical Sciences, Mashhad, Iran; 2 Department of Pharmaceutics, School of Pharmacy, Mashhad University of Medical Sciences, Mashhad, Iran; 3 Department of Pharmaceutical Biotechnology, School of Pharmacy, Mashhad University of Medical Sciences, Mashhad, Iran; 4 Department of Cardiovascular Diseases, Faculty of Medicine, Mashhad University of Medical Sciences, Mashhad, Iran; 5 Lupin Pharmaceutical Research Center, 4006 NW 124th Ave., Coral Springs, Florida, FL 33065, USA; 6 Pharmaceutics Research Laboratory, School of Life Sciences, University of Sussex, Brighton BN1 9QJ, UK

**Keywords:** anti-inflammatory, niosome, NSAIDs, vesicle, non-ionic surfactant

## Abstract

Niosomes are drug delivery systems with widespread applications in pharmaceutical research and the cosmetic industry. Niosomes are vesicles of one or more bilayers made of non-ionic surfactants, cholesterol, and charge inducers. Because of their bilayer characteristics, similar to liposomes, niosomes can be loaded with lipophilic and hydrophilic cargos. Therefore, they are more stable and cheaper in preparation than liposomes. They can be classified into four categories according to their sizes and structures, namely small unilamellar vesicles (SUVs), large unilamellar vesicles (LUVs,), multilamellar vesicles (MLVs), and multivesicular vesicles (MVVs). There are many methods for niosome preparation, such as thin-film hydration, solvent injection, and heating method. The current study focuses on the preparation methods and pharmacological effects of niosomes loaded with natural and chemical anti-inflammatory compounds in kinds of literature during the past decade. We found that most research was carried out to load anti-inflammatory agents like non-steroidal anti-inflammatory drugs (NSAIDs) into niosome vesicles. The studies revealed that niosomes could improve anti-inflammatory agents' physicochemical properties, including solubility, cellular uptake, stability, encapsulation, drug release and liberation, efficiency, and oral bioavailability or topical absorption.

See also the graphical abstract(Fig. 1).

## Introduction

One of the past decade's most attractive drug delivery research fields is drug-targeted delivery with a controlled release rate. Nanocarriers are considered a suitable drug delivery system due to favorable features, such as protecting the active compounds against degradation, reducing toxicity, controlling release, and targeting drug delivery (Parkhe et al., 2018[211]).

Niosomes were first introduced in the 1970s as a carrier for the cosmetic industry (Waddad et al., 2013[291]; Moghassemi and Hadjizadeh, 2014[174]). These structures are vesicles made of one or more bilayers based on non-ionic surfactants (Nasir et al., 2012[190]). In many cases, cholesterol and its derivatives were also used to prepare niosome (Moghassemi and Hadjizadeh, 2014[174]). In the aqueous phase, non-ionic surfactant sheets naturally aggregate to produce concentric bilayer vesicles that can load hydrophilic and lipophilic substances (Sankhyan and Pawar, 2012[247], Patil and Jadhav, 2014[213]).

Many types of research have been conducted on the use of niosomes as drug carriers, and in various studies, it has been observed that niosomes as a carrier play an important role in the treatment of fungal diseases, wound healing, rheumatoid arthritis (RA), psoriasis, and other inflammation-related disorders (Marianecci et al., 2012[163]; Osanloo et al., 2018[199]; Farmoudeh et al., 2020[84]; Qiu et al., 2021[224]; Raja et al., 2022). In addition, Manosri et al.*,* suggested that niosomes can be used as carriers for gene delivery through the skin. Contextually, the transactivator of transcription (TAT, GRKKRRQRRRPQ) peptide has been evaluated in transdermal drug delivery using niosomes on mouse skin. In this study, transdermal absorption and stability of tyrosinase plasmid increased (Manosroi et al., 2013[157]). In another study, pergolide, an anti-Parkinson disease therapy, was examined for its penetration through human skin. It was found that the penetration dependent on the pH of the carrier, and the pH of niosomal formulation can be a determining factor for transdermal drug delivery (Ge et al., 2019[93]).

Due to the physicochemical properties of niosomes, they can load a large number of medicines and increase the bioavailability of drugs by passing through different body barriers, such as the skin and gastrointestinal (GI) barriers (FDA, 2023[87]).

Regard to inflammation-related diseases, autoimmune disorders such as Rheumatoid arthritis (RA), Psoriasis and Intestinal Bowel Syndrome (IBD) affect the large population of patients (Parisi et al., 2020[209], Almutairi et al., 2021[17], Caviglia et al., 2023[49]). For example, in the case of RA, its reported worldwide occurrence from 1980 to 2018 was about 460 per 100,000 individuals in the population (Almutairi et al., 2021[17]). Nonsteroidal anti-inflammatory drugs (NSAIDs) are commonly utilized in the field of rheumatology due to their efficacy as anti-inflammatory and analgesic agents. The symptomatic management of various rheumatic conditions characterized by chronic musculoskeletal pain and various types of acute pain is extensively supported by the use of NSAIDs (Crofford, 2013[61]). Many studies have revealed that transdermal delivery of NSAIDs could decrease adverse effects (AEs), such as GI discomfort, bleeding, and ulceration that would happen in oral NSAIDs (Abdel-Rahman et al., 2013[9]). In these cases, niosomal carriers showed promising applications with the lowest AEs. In addition, niosomes are ideal drug delivery systems for skin disorders, especially with inflammatory regions, such as acne vulgaris and psoriasis (Habib et al., 2022[102]). Psoriasis is accompanied by thickening of the stratum corneum layer of the skin (Orsmond et al., 2021[198]). In this respect, dermal and transdermal drug delivery could be faced with limitations. However, nanocarriers such as niosomes showed the capability of passing through the epidermis (Tambe et al., 2021[275]). Moghddam et al.*,* optimized diacerein-loaded niosomes for management of psoriasis. In their study the images obtained from confocal laser scanning microscopy of diacerein niosomes demonstrated their efficacy in permeating diacerein to a depth of 180 μm. Especially, the fluorescence intensity was extremely high amidst 30 and 90 μm (i.e., the epidermis layer depth) and relatively lower up to 180 μm (i.e., the underlying dermis depth). As such, the diacerein niosomes exhibited a strong capacity to transport diacerein to the viable epidermis layer beside a small quantity of the drug penetrated the dermis layer. The findings confirmed that the niosomes drug delivery system accentuated the penetration of diacerein to the epidermis layer, which is consistent with the results of the *in vitro* skin penetration study (Moghddam et al., 2016[175]). 

In comparison with lipid based nanocarriers like liposomes, SLNs and NLCs, non-ionic surfactants which compose the major contents of niosome bilayers are less susceptible to oxidation and hydrolysis compared to lipids. In this regard they are more stable and they don't need any excess operation like using inert gases during manufacturing process. Non-ionic surfactants are easily available and have low cost in comparison with lipids and polymers which could reduce the costs of final products (Basiri et al., 2017[33]; Chen et al., 2019[55]). Topical application of niosomes alone is restricted due to its liquid nature, causing it to leak from the application site. To overcome this challenge, gelling agents can be incorporated into niosomal dispersion to form a niosomal gel. The use of niosomal gels can provide a reservoir of drugs in the stratum corneum (SC) for sustained release, leading to high drug accumulation in the dermis and epidermis (Chen et al., 2019[55]). Although niosome based products are available in cosmetic market however till now, we have no approved niosomes for medicinal manners. In addition, reported clinical trials on these platforms are limited which are given in Table 1(Tab. 1). Besides that, these platforms are growing up rapidly and nowadays elastic niosomes are presented as promising platforms for topical drug delivery which termed as “Spanlastic” with using edge activators (Ibrahim and Abd-Allah, 2022[114]). 

In the current paper we used databases and search engines such as PubMed, Scopus, and Google scholar, with the keywords “niosome” and “inflammation or anti-inflammatory” from January 1^st^, 2012 to March 2023 and classified all related reports into chemical and natural compounds. Then we described the efficacy of their niosomal formulation based on their *in vitro* and *in vivo* experiments, with considering the type of surfactants, additives and methods of preparation.

There are some disadvantages to niosome's formulation in addition to its advantages, such as its low physical stability, its high probability of aggregation, fusion and percentage of drug loading, as well as the possibility that it will hydrolyze under storage conditions.

## Description and Structure of Niosome

Niosomes are self-assembling vesicles that are made of non-ionic surfactants (Mahale et al., 2012[152]; Marianecci et al., 2014[159]). They can capture hydrophilic cargos in an aqueous core, and lipophilic compounds could be entrapped into the bilayer domains (Abdelkader et al., 2014[3]).

The non-ionic surfactants, which are the main component of niosomes, are cheap, non-toxic, and well-tolerated in both external and internal administration (Bendas et al., 2013[34]; Waheed et al., 2022[292]). Niosome bilayers are more stable than liposomes, especially against oxidation. They need no excessive manufacturing procedures to protect them from oxidation and have higher shelf life than liposomes (Paecharoenchai et al., 2013[200]). Furthermore, niosomes showed great stability in the GI tract when administered orally (Varshosaz et al., 2003[289]; Pardakhty et al., 2007[208]; Marinho et al., 2021[164]). Nowadays, niosomes have attracted many reserachers to emplpy it in the design of novel drug delivery systems for the oral delivery of chemical and natural compounds. Particularly they showed that they could be a good choice for delivery of peptides and proteins, which tend to decompose by extreme variations in pH and the effects of digestive enzymes in the GI tract (Varshosaz et al., 2003[289]; Pardakhty et al., 2007[208]; Sharma et al., 2021[256]). Based on the structure, niosomes are spherical vesicles consisting of one or more bilayers and based on their size, they could be categorized into four categories as follows.

**Figure 2:**
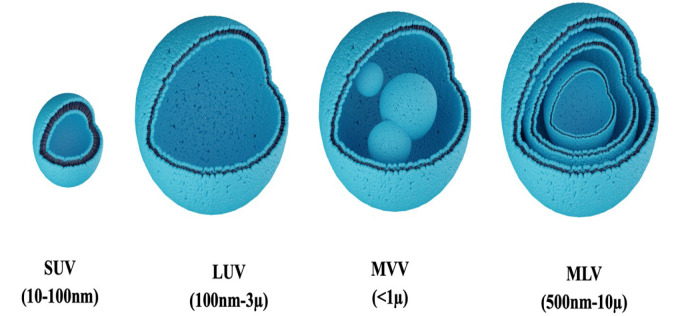
Niosome categories based on size

- Small unilamellar vesicles (SUVs) with a size between 10 to 100 nm,
- Large unilamellar vesicles (LUVs) with a size between 100 nm to 3 µm,
- Multi-lamellar vesicles (MLVs) which have more than one bilayer in size between 500nm to 10 µm
- Multi-vesicular vesicles (MVVs) defined as niosomes with an outer vesicle, which contain smaller vesicles inside, and generally, their size is more than 1 µm (Figure 2(Fig. 2)) (Moghassemi and Hadjizadeh, 2014[174]; Cerqueira-Coutinho et al., 2016[50]; Yasamineh et al., 2022[303]).

## Composition of Niosomes

Niosomes are mainly composed of non-ionic surfactants like Spans and Tweens. In most formulations, cholesterol has been used along with surfactants to increase the stability of bilayers in the same way as liposomes. However, sometimes charge inducer molecules may be added in bilayer composition to change the surface charge of niosomes and also could have unique applications, for example, in cationic niosomes that are used for gene delivery in which nucleic acids that are negatively charged could bind to the bilayers with electrostatic interaction (Grijalvo et al., 2019[100]).

### Non-ionic surfactants

**Figure 3:**
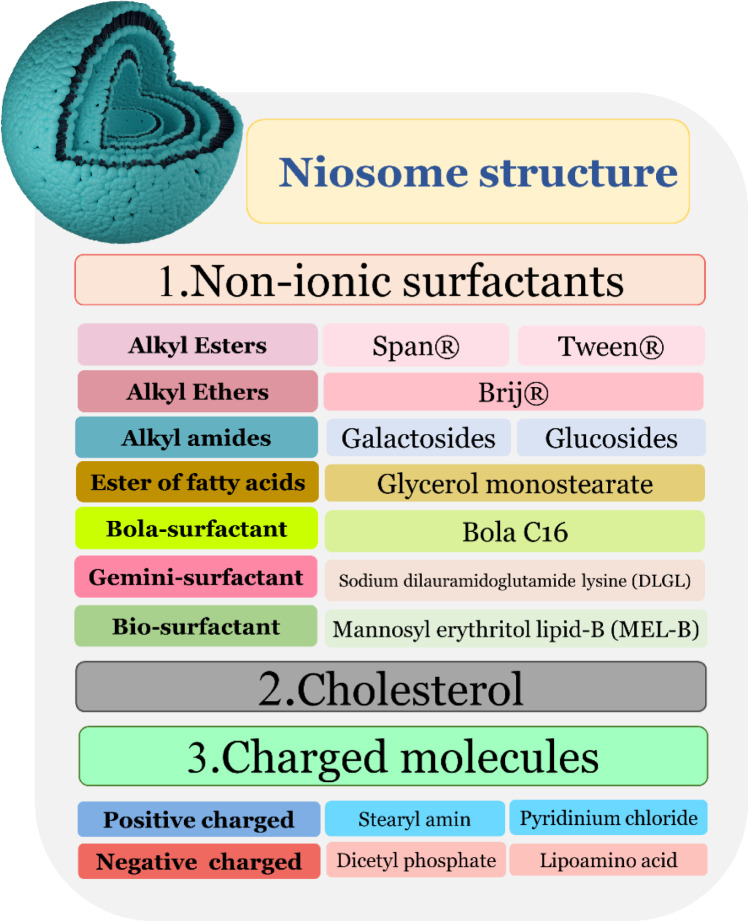
An overview of niosome compositions

Non-ionic surfactants are the major ingredient in the fabrication of niosomes. They have amphiphilic characteristics with a non-polar tail and a hydrophilic head. Compared to cationic, anionic, and amphoteric surfactants, non-ionic do not have any charge, making them more biocompatible and less toxic. Non-ionic surfactants could be employed as emulsifiers and wetting agents in order to increase the solubility and permeability of bilayers. Because of the non-ionic surfactants' amphipathic properties by optimizing the hydrophilic and lipophilic ratio, niosomes could be assembled automatically in aqueous media. The non-ionic surfactants are classified into four major categories: Alkyl esters (Span and Tween), Alkyl amides, Alkyl ethers (Brij), and esters of fatty acids, which are explained in Figure 3(Fig. 3). There are also some new generation of surfactants like bola-surfactants, gemini-surfactants and bio-surfactants (Terayama et al., 2023[279]). Hydrophilic-lipophilic balance (HLB) and critical packing parameter (CPP) are two important parameters in choosing the proper surfactant.

**Figure 4:**
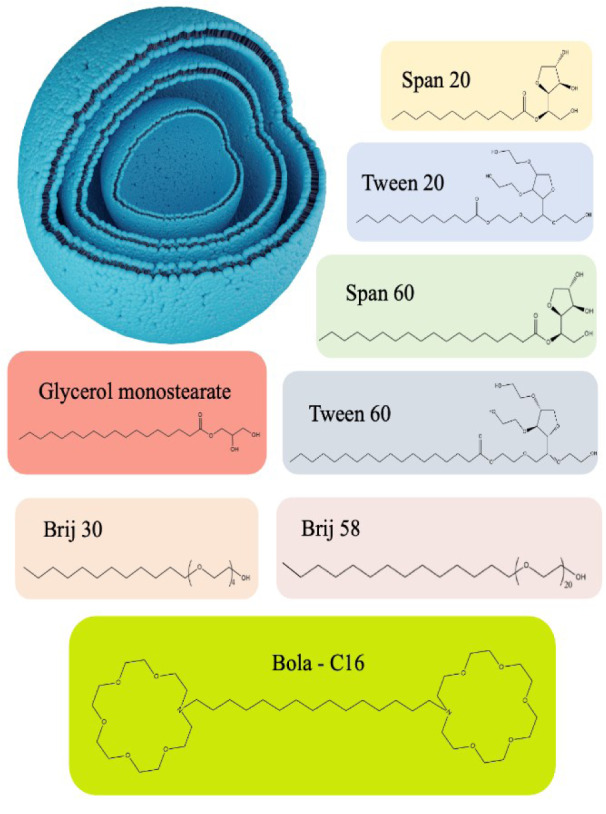
Most frequent surfactant used in niosome preparation with chemical structures

Furthermore, the HLB value is the critical parameter in improving drug entrapment efficiency (EE), and it should be optimized accurately for the preparation of niosomes. Besides, the surfactant's CPP must be in the range of 0.5 <CPP<1 to produce the bilayers; out of this range, surfactants tend to form micellar structures (Moghassemi and Hadjizadeh, 2014[174]). Therefore, novel surfactants have been made in order to create innovative niosomal systems. These surfactants are composed of two identical aza-crown ether units linked by a long alkyl chain and responding as polar heads known as bola-form amphiphiles (Figure 4(Fig. 4)). When they come into contact with cholesterol, they can form colloidal systems. Novel niosomes were created by using hexadecyl-bis-(1-aza-18-crown-6) (Bola C16), Span 80, and cholesterol (2:5:2 M ratio), especially for topical and transdermal delivery of 5-fluorouracil (5-FU) in skin cancer (Paolino et al., 2007[207], 2008[206]).

### Cholesterol

One of the most important additives that are used in niosomal formulations is cholesterol. Cholesterol plays an important role in the stability of niosome membrane (Manosroi et al., 2003[156]; Pozzi et al., 2010[218]). Cholesterol influences the physical properties and structure of niosomes, possibly by its interaction with nonionic surfactants. In many cases adding cholesterol could increase the rigidity and stability of bilayers (Nasseri, 2005[191]). Whether to add cholesterol in formulation or not depends on the surfactant's HLB. As HLB rises above 10, more cholesterol must be proposed to account for the larger head groups. In Brij76 (HLB 12.4) niosomes, minoxidil entrapment, was enhanced by increasing cholesterol content, but not in Brij 52 (HLB 5.3) niosomes. Some experiments showed that the size of niosomes would increase by increasing the cholesterol content in the formulation (Abdelkader et al., 2014[4]). However, in general, we cannot predict the effect of cholesterol on non-ionic surfactants, and there is no general trend. To obtain the optimized formulation, some experimental design might be required.

### Charge inducers

Charge-inducing agents stabilize bilayer membranes by making the niosome surfaces negatively or positively charged, preventing aggregation by electrostatic forces (Khan et al., 2016[131]). In order to prepare niosomes, negatively charged ionic compounds such as dicetyl phosphate (DCP) and positively charged compounds such as stearyl pyridinium chloride or stearyl amine (STR) are frequently employed as charge-inducing agents. As a general rule, these charged molecules are incorporated into formulations in an amount ranging from 2.5 to 5 mol % (Mokhtar et al., 2008[182]; Moghassemi and Hadjizadeh, 2014[174]). In any case, exceeding the defined limit of the charge-inducing molecule can avoid the formation of niosomes (Moghassemi and Hadjizadeh, 2014[174]).

## Methods of Preparation

Several methods exist to fabricate niosomes; the preparation process is an important factor in the size and structure of final niosomes. For example, several studies are using TFH and heating method results in MLVs. On the other hand, SLVs can be prepared by EIM and microfluidization. Some of the widely used methods for preparing niosomes are discussed in the following.

### Thin-film hydration method (TFH)

Thin-film hydration method (TFH) or “Hand Shaking Method” is mainly used to fabricate niosomes. An organic solvent is added to a round-bottom flask, and then cholesterol and surfactants are added. In a vacuum rotary evaporator, the organic solvent would be evaporated; then, a thin layer of surfactant and cholesterol forms on the inner glass flask after evaporation. Phosphate buffer or deionized water would be used to hydrate the layer further. This method could give us MLVs (Rajera et al., 2011[230]; Gandhi et al., 2012[91]; Moghassemi and Hadjizadeh, 2014[174]).

### Freeze and Thaw method

The freeze and thaw method employs niosomes made with the TFH process. Niosomal suspension (8 ml) is frozen in liquid nitrogen for one minute and then thawed in a water bath at 60 °C for a further minute. Utilization of this method results in MLVs (Abdelkader et al., 2014[3]; Moghassemi and Hadjizadeh, 2014[174]).

### Solvent injection method

This method combines cholesterol and a surfactant in a mutual organic solvent such as diethyl ether. This combination is gradually added to a hot aqueous media that contains the medication. This solution's temperature is maintained at or above 60 °C (Tc) (Marwa et al., 2013[165]).

### Sonication method

In a buffered drug solution, a mixture of cholesterol and surfactant is added then at 60 °C, a probe sonicator is used to sonicate the mixture. Utilization of this method results in MLVs (Bansal et al., 2013[29]).

### Microfludization method

Surfactants and ingredients are dissolved together and then delivered under pressure from a reservoir flask into an ice-filled interaction chamber. The solution is moved through a cooling chamber so any heat generated in the operation process can be expelled. This method could produce SUVs (Kazi et al., 2010[128]; Kumar and Rajeshwarrao, 2011[134]; Moghassemi and Hadjizadeh, 2014[174]).

### Reverse phase evaporation method (REV)

Surfactant and cholesterol are combined in an organic solvent. The aqueous phase also is prepared for the water-soluble drug. The two phases are combined and homogenized. The organic phase is eliminated under negative pressure. This method results in LUVs (Kumar and Rajeshwarrao, 2011[134]).

### Heating method

In the buffer solution, the cholesterol and surfactant are hydrated separately. First, 120 °C of heated hydrated cholesterol mixture is applied for one hour. Then, surfactants and other ingredients are incorporated while the temperature is lowered while continuously stirring. This method produces MLVs (Kazi et al., 2010[128]; Mahale et al., 2012[152]).

### Transmembrane pH gradient method

In an organic solvent, such as chloroform, surfactant and cholesterol are dissolved in an equal ratio. A thin lipids coating is created on the inner surface of the flask as a result of the organic solvent being withdrawn under reduced pressure. The layer is hydrated, utilizing vortex and citric acid. The mixture that results is freeze-thawed. This method could give us MLVs. Notably, a di-sodium hydrogen phosphate solution can be used to regulate the pH (Kazi et al., 2010[128]; Kumar and Rajeshwarrao, 2011[134]).

### Supercritical carbon dioxide fluid

Supercritical carbon dioxide (scCO_2_) is a safe, non-flammable, and inexpensive solvent that is utilized instead of organic solvent to produce vesicles. Simple operating conditions of scCO_2_ are significant for encapsulating water-soluble and thermally labile compounds in niosomes. Sometimes a co-solvent like ethanol could be employed to improve the EE of various water-soluble compounds in the bilayers (Manosroi et al., 2008[155]).

### Microfluidic chips

Microfluidics is an interdisciplinary field encompassing the science of effectively handling fluids with volumes that range from microliters to nanoliters or even smaller quantities, in channels possessing dimensions in the range of tens to hundreds of micrometers. Its versatile potential has been widely recognized and utilized in various fields due to the facile manipulation of solutions or reagents, enabling numerous advantages over conventional chemical routes. Additionally, microfluidic devices enable the preparation of nanomaterials with controlled shape, size, morphology, and composition due to their effective mixing and rapid chemical reaction (Tian et al., 2022[280]). During the COVID-19 pandemic, microfluidic systems were effectively utilized in the development of mRNA-based vaccines for the SARS-CoV-2 virus (Lopes et al., 2022[147]). Niosomes prepared with microfluidic chips showed good homogeneity and lower size and PDI in comparison with conventional TFH method (Yeo et al., 2018[304]).

A microfluidic chip method provides products with a smaller size and a higher uniformity, and the rate of drug loading with this method is higher than other methods. This method is also scalable, but may damage lipids during production due to high pressures. Using sonication, particles are quickly reduced in size, but metal particles may escape from the device probe and cause pollution. The solvent injection method dissolves lipids quickly and the percentage of lipids in our formulation is high, but the organic solvent may remain in the final product and contribute to the degradation of lipids over time (Umbarkar, 2021[287]).

## Proniosome

A thin coating of a non-ionic surfactant is applied on a water-soluble carrier to create a proniosome. To make proniosomes, the water-soluble carriers must be non-toxic, safe, and highly soluble in water to facilitate hydration. Proniosomes have been produced using maltodextrin, sorbitol, mannitol, glucose, lactose monohydrates, and sucrose stearate (Marianecci et al., 2014[159]; Yuksel et al., 2016[307]; Gao et al., 2020[92]). Proniosomes, available in a dry powder form, are different from normal niosomes in ways such as being more stable, less likely to form aggregates, and less likely to leak drugs. There are several ways to make proniosomes, including the slurry approach, the slow spray coating method, and the coacervation phase separation method. Depending on the preparation technique, they can be found in two different forms, namely liquid crystalline proniosomes and dry granular proniosomes (Abd-Elbary et al., 2008[1]; Mokhtar et al., 2008[182]; Ibrahim and Shehata, 2012[113]; Elhissi et al., 2013;[71] Csongradi et al., 2017[62]).

## Spanlastics

Spanlastics are new vesicular carriers that include an edge activator in their composition, also known as "modified niosomes". Spanlastics typically have sorbitan esters and edge activators such as Tweens (Farghaly et al., 2017[82]). The term Spanlastic (Span + Elastic) was first used in 2011. Kakkar and Kaur made ketoconazole-loaded spanlastics for eye drug delivery using Span 60 and Tween 80 (Kakkar and Kaur, 2011[122]). Unlike niosomes, they do not contain cholesterol in their structural configuration. Niosomes, on the other hand, typically have around 10-40 % of cholesterol. In general, spanlastics exhibit a similar relationship to niosomes as transferosomes do to liposomes. The addition of surfactant to liposomes makes them deformable. Likewise, non-ionic surfactant (without cholesterol) makes spanlastics ultra-deformable (Farghaly et al., 2017[82]). In contrast to a drug solution, these deformable vesicular carrier systems demonstrate superior permeability (Witika et al., 2021[298]). Spanlastics have been purposefully designed to achieve targeted action at specific sites. The remarkable elasticity of these vesicles empowers them to effortlessly traverse the corneal membrane, enabling them to access both the anterior and posterior segments of the eye with precision to target the retinal pigment epithelium, vitreous cavity, and choroid (Kumar et al., 2013[133]; Elazreg et al., 2015[70]). Spanlastics offer many benefits including ease of use, precise targeting, stability, and strong adherence by patients (Liu et al., 2019[146]). Spanlastics possess great potential as vesicles for delivering antifungal drugs, owing to the edge activator they contain. This activator offers tremendous flexibility, thereby enhancing drug permeability (Mosallam et al., 2022[184]). Spanlastics have various applications in the field of site-specific drug delivery, including ocular delivery, oral delivery, topical delivery, nasal delivery, otopical delivery, ungual delivery (Chauhan and Verma, 2017[53]).

Edge activators: These surfactants possess a distinct characteristic of elevated hydrophilicity, also known as the HLB value. These surfactants possess only a solitary chain. Edge activators refer to certain components that have the ability to soften the bilayer. These components are typically biocompatible surfactants, to which an amphiphilic substance is added to further enhance the lipid bilayer's permeability and flexibility. It enhances the flexibility of the bilayer by reducing the interfacial tension between them. Edge activators tend to generate larger spherical vesicles, which in turn lead to smaller particle sizes. Tween 80 serves as an edge activator which enhances the elasticity of vesicles. Any vesicle that exceeds the biological membrane's pore size can effortlessly move from the exterior to the interior due to the temporary enlargement of pore size caused by Tween 80. Moreover, it enhances drug penetration and increases drug concentration inside the vesicle. Hydrophilic surfactants can disturb vesicular membranes, increase their flexibility, and cause varying levels of packing disruption (Kamath et al., 2023[123]). The nanovesicular carriers' features are enhanced through the use of ethanol. Its ability to condense membranes renders it quite valuable. It facilitates the improvement of drug entrapment and partitioning within the vesicles. The thickness of the vesicular membrane has been decreased, thereby augmenting the entrapment capability of drugs by the spanlastic system. Furthermore, altering the net charge of the system to a negative zeta potential serves to stabilize the steric effect to a certain degree (Kamath et al., 2023[123]).These vesicles based on spans could prove to be a significant breakthrough in enhancing the retention of bioactive substances within the eye. Furthermore, there is potential for these vesicles to be utilized in conjunction with various other drug classes, including anti-HIV, anti-angiogenetic, oligonucleotides, and anti-vascular endothelial growth factors (Lalu et al., 2017[141]).

## Niosome and Anti-Inflammatory Agents

### Chemical compounds

#### Non-steroidal anti-inflammatory drugs (NSAIDs)

##### Aceclofenac

Aceclofenac is a NSAID drug that has poor solubility in water, but is known for its ability to act specifically on inflammatory sites and reduce inflammation. It is highly effective in treating various inflammatory conditions such as rheumatoid arthritis, osteoarthritis, and ankylosing spondylitis (Trivedi et al., 2008[283]). However, despite its therapeutic benefits, prolonged oral consumption of aceclofenac can lead to several gastrointestinal side effects. With a lower molecular weight (354.1) and a relatively short half-life (4-4.3 hours) in plasma, aceclofenac has the potential to be administered transdermally (Trivedi et al., 2008[283]).

Numerous researchers have diligently investigated aceclofenac niosomes (Solanki et al., 2010[267]; Srinivas et al., 2010[269]; Mishra et al., 2014[173]; Sammour et al., 2019[246]). For instance, Solanki et al.*,* have developed and fine-tuned aceclofenac niosomes for transdermal administration, with the intention of enhancing drug permeation over a prolonged duration (Solanki et al., 2010[267]). Niosomes were prepared using the TFH method. The chosen batches of niosomes were then incorporated into a carbopol gel matrix to create niosomal gel formulations. These formulations were then evaluated for *in vitro* release, skin permeation, and *in vivo* studies. Each of the niosomal gel formulations that were prepared showed a significant improvement (P<0.05) in the cumulative amount of drug permeated, steady state transdermal flux, and an increase in paw thickness. Among these formulations, NA6, which was prepared at a high level of molar ratio of drug to lipid and a medium level of volume of hydration medium, showed the best permeation and effectiveness. This may be due to efficient hydration of the film and a greater total amount of drug entrapped. The study demonstrates that niosomal gel formulations have the potential to offer a promising transdermal delivery of aceclofenac to improve efficiency and better patient compliance.

##### Celecoxib

Celecoxib is a frequently prescribed medicine for RA and is available on the market in the dosage forms of tablets, capsules, and oral suspension. It has a variable absorption pattern and takes place 3 to 4 hours after oral administration (Moghimipour et al., 2015[176]; Jafari et al., 2023[117]). Additionally, celecoxib has plasma clearance and is affected by hepatic first-pass metabolism (Abdelrahman et al., 2017[8]). Therefore, when used for an extended period, celecoxib and other cyclooxygenase-2 (COX-2) inhibitors can cause cardiotoxicity. Modified delivery strategies have been developed to boost the therapeutic effects of celecoxib and lessen the occurrence of AEs. Shakeel et al.*,* stated that the utilization of a transdermal nanoemulsion formulation increased the bioavailability of celecoxib. Besides this study, Moghimipour et al.*,* suggested that celecoxib-loaded liposomes could be used as a transdermal drug delivery method (Shakeel et al., 2008[254]; Moghimipour et al., 2015[176]). Celecoxib-loaded PEGylated liposomes have increased anti-arthritic efficacy, according to Dave's study (Dave et al., 2019[67]). Other celecoxib-loaded nano-carriers showed that these drug delivery systems could enhance the cytotoxic, anti-arthritic, anti-inflammatory, and pain-relieving effects of celecoxib (Kaur et al., 2007[127]; Perlstein et al., 2014[215]; Auda et al., 2015[25]; Refaat et al., 2019[236]; Alaaeldin et al., 2021[15]).

Alaaeldin et al.*,* investigated celecoxib niosomes and spanlastics with the spraying method. Their results showed that the percentage reduction in edema in the treatment groups of rats was significant compared to the control group (Alaaeldin et al., 2021[15]). Following seven days of treatment, the percentage of edema reduction after day 30 of arthritis induction was comparable between the groups treated with celecoxib gel and diclofenac sodium (DS) gel (42.13 ± 2.2 % and 40.25 ± 4.3 %, respectively). However, celecoxib-loaded niosomes reduced edema size by up to 64.1 %. The spraying technique/ modified injection method, which was mentioned earlier, was used to produce spanlastics (SP) (Refaat et al., 2019[236]). Celecoxib-loaded SP also showed great promise in reducing edema size up to 73.45 % (Alaaeldin et al., 2021[15]).

In comparison to healthy rats, RA-induced rats had higher overall expression levels of nuclear factor kappa-light-chain-enhancer of activated B cells (NF-кB), tumor necrosis factor-alpha (TNF-α), and COX-2. In contrast, the levels of TNF-α, NF-кB and COX-2 were dramatically decreased in celecoxib and diclofenac-treated animals compared to the arthritic control rats. NF-кB is a crucial transcription factor in cells that regulates the production of several cytokines and is crucial for the development of RA. According to reports, NF-кB regulates the production of TNF-α, whereas TNF-α acts as an upregulation of NF-кB activation. As a result, TNF-α inhibition reduced all pro-inflammatory cytokines whose production depends on NF-кB. The overall expression levels of NF-кB, TNF-α, and COX-2 were higher in the rats with RA than in the healthy rats (Tables 1(Tab. 1) and 2(Tab. 2)) (Taylor et al., 2009[277]; Fattahi and Mirshafiey, 2012[86]; Cheng et al., 2015[56]).

##### Diclofenac sodium

The long-term management of RA, osteoarthritis (OA), and ankylosing spondylitis has so far been accomplished with the help of diclofenac sodium (DS), a potent NSAID with noticeable analgesic properties. Since diclofenac's biological t_1/2_ is only 1 to 2 hours, and the liver significantly metabolizes it, frequent administration is required (Liu et al., 2010[144]; Akbari et al., 2022[14]; Jafari et al., 2023[117]). It is viewed as a perfect drug for controlled transdermal drug delivery because of its relatively short biological half-life and AEs (Kienzler et al., 2010[132]; Akbari et al., 2022[14]).

Akbari et al.*,* investigated the formulation and effectiveness of niosomal diclofenac in an animal model. In this study, DS, a hydrophilic medicine, was successfully integrated into niosomes made of a mixture of cholesterol and surfactants for transdermal delivery.

DS was loaded into niosomes in an amorphous form without chemically interacting with the other niosome components, with EE of 47.16 ± 1.60 %. In animal models, niofenac (niosome of diclofenac) significantly reduced pain and inflammation compared to the control group (Akbari et al., 2022[14]).

Additionally, Marwa et al.*,* looked into how different surfactants affected the loading and release of diclofenac niosomes. Using co-surfactants, such as various types of Tweens with cholesterol in a molar ratio of 25:25:50, increased the EE %. Compared to niosomes made solely from Span 60 and cholesterol, those made from a combination of Span 60 and Tweens demonstrated a more considerable reduction in the *in vitro* release of diclofenac niosomes and produced less leaky niosomes. This outcome might be explained by the co-surfactant-induced increase in membrane stiffness and decrease in permeability (Marwa et al., 2013[165]).

According to Naresh et al., DS-encapsulated niosomes were found to have anti-inflammatory effects in arthritis-inflicted rats*.* Furthermore, it was discovered that the niosomal formulation made of Tween 85 produced more steady anti-inflammatory activity for more than 72 hours after administration of a single dose (Naresh et al., 1994[189]).

The ability of manufactured niosomes to penetrate the skin more effectively than the drug solution was also demonstrated by Tavano et al.*,* The improved influence of niosomes on the *ex vivo* cutaneous uptake of DS was evaluated by means of Franz diffusion cells. Diclofenac solution's permeation through the skin was less than that of all niosomal formulations, proving that niosomes are percutaneous permeation enhancers. The findings revealed that those formulations with the drug placed in the aqueous core had the highest cumulative levels of diclofenac that permeated rabbit skin after 24 hours (Tavano et al., 2014[276]).

##### Flurbiprofen

Flurbiprofen (FBP) is one of the phenyl alkanoic acid derivatives of NSAIDs, which has recently been extensively studied in the treatment of ophthalmic diseases. Flurbiprofen is a polar molecule and is slightly soluble in water (logP=4, solubility: 0.013 mg/mL). It effectively treats eye inflammation after surgery and during cataract surgery (Brown and Roberts, 1996[42]; Ahuja et al., 2008[12]).

Recent studies have shown that FBP can also reduce bacterial infections caused by contact lenses (Bandara et al., 2004[28]). Due to our limitations in ocular drug delivery, only about 5 % of the drug can pass through the cornea and penetrate the tissues inside the eye. To overcome this limitations, various formulations of FBP have been prepared (Zhang et al., 2004[309]).

Various formulations, including polycaprolactone nanoparticles (Ramos Yacasi et al., 2016[233]), FBP complex, and EudragitE100 (Quinteros et al., 2014[225]), solid lipid nanocarriers prepared with Compritol^®^888 (Gonzalez-Mira et al., 2012[98]), have been designed and investigated to improve drug diffusion throughout the cornea. One of the most effective approaches to increase drug effectiveness and protect the drug from being metabolized by tear enzymes is to trap the drug in two-layered structures of amphiphilic molecules such as niosome (Kaur et al., 2004[126]; Kumar and Rajeshwarrao, 2011[134]).

El-Sayed et al., prepared FBP niosomes to increase the bioavailability of the drug in the eye (El-Sayed et al., 2017[77]). FBP niosomes are used once or twice a day due to prolonged contact with eye tissues, which is one of the advantages of niosomal formulations over standard drops; hence, the patient's compliance would be increased. El-Sayed et al.*,* first prepared FBP-loaded niosomes, then loaded the whole formulation into a gel to increase viscosity for ophthalmic application. In the above study, Span 60 was chosen as the surfactant, and ratios of 1:1 for cholesterol to Span and 1:2 ratios for the drug to surfactant was selected as the most appropriate ratios in the preparation of niosome. Pharmacokinetic studies were performed on albino rabbits. Surface surfactants with high phase transition temperature created niosomes with higher loading capacity (Abdelkader et al., 2010[5]). Since FBP is a hydrophobic drug, formulation stability has a direct relationship with the ratio of cholesterol; as the cholesterol content increases, more drug is placed between the two layers, and so the stability of the formulation increases (Uchegbu and Vyas, 1998[286]). It has been reported in various studies that the highest drug loading occurs at a concentration of 30 % mol/mol of cholesterol (Uchegbu and Vyas, 1998[286]; Makeshwar and Wasankar, 2013[154]).

As the cholesterol concentration increases, lipid bilayer width also increases, and therefore the drug loading increases (Mohawed et al., 2014[181]). The release study showed that the drug could be released in two stages. First, it was released from the niosome, then dispersed in the gel network, and the second release stage was observed, which creates a delayed release. The C_max_ of FBP niosomes was high (1366.68 ± 36.39 ng/ml), indicating that the drug's absorption was increased in the eye's aqueous media.

There was an order of increasing residence time on the cornea according to the following sequence: gel loaded with F5 > dispersion of F5 >> FBP solution, with the niosome at the base of the gel being removed from the eye later than the rest of the gel. The FBP niosomes remained in the eye for 12 hours. As a result, FBP niosomes effectively met the study's objectives by successfully maintaining the drug concentration in the rabbit's aqueous humor, eliminating the need for repeated injections, and thus increasing patient compliance. Several studies have demonstrated that niosomes are a viable drug delivery mechanism to improve drug penetration into the eye and boosting drug bioavailability.

##### Ibuprofen

Ibuprofen is another phenyl propionic acid derivative with analgesic and antipyretic properties that are used to treat RA, ankylosing spondylitis, OA, and other inflammatory conditions (Jafari et al., 2023[117]). Unfortunately, half-life of ibuprofen in the body is relatively short, and to maintain the effective concentration of the drug, we need to repeat the medication intake, which can cause many side effects. Thus, developing a site-specific drug delivery system for ibuprofen seems necessary in order to prevent side effects (Mujeeb and Sailaja, 2017[186]; Irvine et al., 2018[115]).

Based on Kumar's article, nanoparticles made of methacrylate copolymer increased the duration of effect and anti-inflammatory activity twofold (Kumar et al., 2003[136]). Furthermore, Esmaeili et al.*,* also showed that nanoemulsion-based ibuprofen gel creates more medicine concentration than ibuprofen gel (Table 2(Tab. 2); References in Table 2: Akbari et al., 2022[14]; Alaaeldin et al., 2021[15]; Aziz et al., 2018[26]; Bhardwaj et al., 2022[35]; El-Mahdy et al., 2020[72]; Elmehy et al., 2021[73]; El-Say et al., 2016[76]; Erfani-Moghadam et al., 2020[78]; Habib et al., 2022[102]; Hatem et al., 2018[107]; Jigar et al., 2011[120]; Kumbhar et al., 2013[137]; Kushnazarova et al., 2021[138]; Marwa et al., 2013[165]; Marzoli et al., 2019[166]; Moghddam et al., 2016[175]; Mohamed et al., 2021[177]; Mohammadi et al., 2017[178]; Mohanty et al., 2020[180]; Noor and Rajab, 2022[193]; Priprem et al., 2014[221]; Shah et al., 2021[252]; Shilakari Asthana et al., 2016[260]; Tavano et al., 2014[276]; Wongsuwan et al., 2020[299]) (Marianecci et al., 2013[162]; Esmaeili et al., 2022[79]).

Marianecci et al. (2013[162]) developed a formulation of ibuprofen-cyclodextrin complex-loaded niosomes to partially address this issue because ibuprofen is a lipophilic medication and is sparingly soluble in water. In addition, numerous lipophilic drugs' solubility and rate of dissolution have been enhanced with the help of cyclodextrins by including complexation in their hydrophobic cavity.

The duration of drug release as well as the penetration of the drug via the synthetic membrane, was much better for formulations containing cyclodextrin. The drug release profiles of the ibuprofen niosomes, which were made with cholesterol and the non-ionic surfactants Tween 60, Tween 65, and Span 60, were promising and depended on the molar ratio of the cholesterol: surfactant (Marianecci et al., 2013;[162] Ghanbarzadeh et al., 2015[95]).

It is acceptted that some pathological states have pH profiles that differ from those of tissues in normal states. For example, ischemia, infection, inflammation, and cancer are some conditions frequently linked to acidosis. Rinaldi et al.*,* created pH-sensitive niosomes for transdermal ibuprofen delivery by combining Tween 20 and its derivatives with glycine (Rinaldi et al., 2017[239]).

Niosomes that had been drug-loaded underwent several tests for *in vivo* antinociceptive and anti-inflammatory activity in murine models. The pH-sensitive niosomes were shown to be safe and stable in the vicinity of fetal bovine serum. This result of using niosomes to make ibuprofen work better as an anti-inflammatory is fascinating for local delivery (Rahmanian-Devin et al., 2021[229]; Shumilov et al., 2010[262])*, *studied Ibuprofen-loaded and pH-sensitive niosomes that can have consistent and long-lasting antinociceptive effects. They showed their formulation's effectiveness in treating acute and chronic inflammation in animal models (Marzoli et al., 2019[166]).

##### Lornoxicam

Lornoxicam (LOR) reduces prostaglandin synthesis through cyclooxygenase inhibition, and its analgesic, antipyretic, and anti-inflammatory actions have been proved. LOR has a short elimination half-life of 3-5 h, in contrast to other oxicams. LOR prevents the cyclooxygenase enzyme from working, which prevents the production of prostaglandins. In the same concentration range, it suppresses both isoforms, i.e., COX-1 and COX-2. Desensitization of peripheral nociceptors has resulted from this, which further reduces inflammation. LOR is prescribed for mild to moderate OA and RA pain and inflammation. It is offered in parenteral formulations (4 mg/ml) for intravenous and intramuscular administration in addition to an oral formulation with an immediate release profile. The daily dose by oral route should not be increased by more than 16 mg. The recommended dose range is from 8 to 16 mg daily (Zhang et al., 2005[310]; Hamza Yel and Aburahma, 2010[103]; Kumbhar et al., 2013[137]).

Kumbhar et al. (2013[137]) studied on niosome based gel of LOR (LOR-Nio gel). They developed LOR-Nio, which contained cholesterol and Span 60 in a 1:3 molar ratio with dicetyl palmitate as the stabilizer in the concentration of 30 M. The anti-inflammatory activity of the LOR-Nio gel was measured in a CA-induced rat paw edema model to assess the LOR-Nio gel's effectiveness *in vivo*. Niosomal gel demonstrated an inhibition of the edema of the rat paw that was 87.69 % higher than that of plain Lor gel (53.84 %) at a significance value of 0.05. These outcomes may be attributable to the formulation's enhanced skin permeation. There are several mechanisms that can be used to explain improved skin permeation and drug deposition from LOR-Nio gel. The formulation's nonionic surfactants enhance permeation. Intracellular lipid bilayers of the skin lose their strength because due to the presence of surfactants in the vesicular form, which improves drug absorption. The LOR's skin permeation is enhanced by its increased solubility. Niosomes' vesicular structure facilitates the drug's permeation and deposition in the skin. Compared to plain aqueous gel, lipid-based vesicles in the nanometer size range demonstrated superior occlusive, enhancing skin hydration and, consequently, drug deposition (Kumbhar et al., 2013[137]).

The promising results in the formulation were also seen in the study of Bini et al.*,* when Span 60 was incorporated into the formulation. Due to its high phase transition temperature and low HLB (Hydrophilic Lipophilic Balance), Span 60 is the best surfactant of all because it produces stable vesicles in an appropriate size. Another aspect of selecting Span 60 was its suitable CPP, which is between 0.5 and 1 for this surfactant, making it proper for producing spherical vesicles (Bini et al., 2012[36]).

Madan et al.*,* developed LOR-loaded niosomes and tested them for their anti-inflammatory properties. In order to prepare this formulation, they employed the TFH technique. The dispersion of niosomes ranged from 0.259 to 0.492, indicating moderate homogeneity. Carbopol was used to transform the formulation into a gel so that it could be used more efficiently for testing (niosomal gel). Wistar rats were used in *in vivo* and *ex vivo* studies to test the formulation's ability to penetrate the skin and reduce inflammation. The formulation with a 1:1 ratio of cholesterol and Span 60 demonstrated higher permeation through rat skin and EE of up to 66 %. Based on their findings, the authors stated that LOR-loaded niosomal gel formulation could be utilized as a drug delivery system (Madan et al., 2016[150]).

##### Naproxen (NPX)

Naproxen (NPX) is a propionic acid-derived NSAID (Ravikumar et al., 1985[235]; Jafari et al., 2023[117]) with analgesic and anti-inflammatory properties. NPX is generally used in the treatment of inflammatory diseases such as RA, OA, acute gout, ankylosing spondylitis, and tendonitis (Todd and Clissold, 1990[282]). It operates by inhibiting both COX-1 and COX-2 (Hawkey, 2001[109]). Its low efficacy is due to its low water solubility (around 15.9 mg/L at 25 °C, log P=3.18) and elimination half-life (12-14 h) (Szura et al., 2014[274]). Regular use of NPX may have several AEs, such as GI discomfort, bleeding, and ulceration (Stefanko et al., 2008[270]). One typical strategy for dealing with these problems is temporarily hiding the carboxylic acid group to decrease its direct impact on the GI tract. The binding of certain biocompatible compounds via the carboxylic acid functional group upon creating an amide bond can accomplish this (Aboul-Fadl et al., 2018[10]). Aboul-Fadl et al.*, *reported novel NPX derivatives with growth-inhibitory effects against human colon carcinoma cells via a COX-independent mechanism. The carboxylic group of NPX is crucial for strong hydrogen bonding at the enzyme active site, according to the crystal structure analysis for NPX-bound COX-2. NPX binding to the COX active site may be reduced by blocking or replacing this moiety. Some studies have shown that sulindac's anticancer activity can be increased through a COX-independent mechanism by carboxylic acid alteration (Whitt et al., 2012[295]; Kumar et al., 2016[135]). 

Kumar et al.*, *examined the nanoformulation of NPX by a new technique known as evaporation-assisted solvent-antisolvent interaction (EASAI). They used three different polymers such as polyvinyl alcohol (PVA), hydroxypropyl methylcellulose (HPMC), and polyvinylpyrrolidone (PVP). They aimed to control the size and morphology of drug particles to boost the anticancer activity of poorly soluble NSAIDs. The results showed NPX-PVP had the most vigorous anti-cancer activity out of all Nano formulations when tested against the Leukemia cancer cell line. NAP-PVP's anti-Leukemia activity was more than twice as strong as doxorubicin, a common cancer medication (Kumar et al., 2016[135]). In the study of Erfani-Moghadam et al., they investigated the ST8MNV NPX nanoformulation (Erfani-Moghadam et al., 2020[78]). This formulation contains Tween 80 and squalene which is very similar to the MF59^® ^(adjuvant of Novartis influenza vaccine), which has been experienced in millions of people with no important immunogenicity or safety issues (O'Hagan et al., 2013[195]). The niosome was fabricated by the TFH method along with sonication. Based on their results, this nanoformulation potentially improved the cytotoxicity of NPX molecules and could increase anti-inflammatory effects. They compared the effect of free NPX and ST8MNV nanocarriers on cell toxicity in numerous cancer cell lines. 

Previously, in the study of Mokhtary et al.*,* they demonstrated that nanovesicles containing 520 micromolar of Tween 80 and 520 micromolar of squalene not only had no significant toxicity but also could promote the growth of MDA-MB-231, MCF-7 cancer cell lines, and normal monocytes. This result could be attributed to the existence of squalene as a natural lipid (Mokhtary et al., 2018[183]). Puras et al.*,* demonstrated that squalene-containing niosomes are safe for ARPE-19, HEK-293 cells, and the rat retina (Puras et al., 2014[223]). In the study by Mohanty et al., they prepared transdermal NPX niosomes by the TFH method. The optimized NPX niosome provided a vesicle size of about 376.12 nm, EE of 86.43 %, and flux was about 27.56 g/cm^2^/h. The *in vitro* release profile and permeation study exhibited prolonged and enhanced drug release. In addition, the anti-inflammatory effect was assessed in animal models. The results of mean edema (%) and inhibition (%) in rats from both treated groups have demonstrated a significant difference (p<0.001) from the control group. 

Compared to the NPX standard gel (NAPSG), optimized NPX niosomes gel (NAPNopt) showed higher inhibition (p<0.001) in paw edema volume at all time points. The percentage of inhibition in NAPNopt-treated group varied from 40.39 % to 70.45 %, while NAPSG-treated rats were only able to reduce through 10.63 % to 32.86 % in 24 hours. It was discovered that the NAPNopt -treated animals had a maximal and more sustained level of inhibition than the NAPSG-treated animals. The study showed NPX had higher solubility in niosome gel and higher concentration that permeated through the skin and entered the systemic circulation. The existence of cholesterol and Span 60 tends to increase NPX encapsulation in niosome gel and raises its anti-inflammatory activity attributed to better skin penetration. The formulation, which had maximum drug release at 6 h, showed maximum NPX permeation via the skin and systemic circulation. These findings revealed that encapsulating NPX in a niosome-based gel enhances its biological activity owing to its high skin penetration potential (Table 2(Tab. 2)) (Mohanty et al., 2020[180]). 

Hiral et al.*,* evaluated the effect of formulation properties on NPX proniosome physicochemical features. Hydrophilic niosome bilayers can increase water uptake and vesicle size. Hydrophilic Tweens could fabricate larger vesicles than hydrophobic Spans. Surfactants with long alkyl chains (like what exists in Tween 80 and Span 80) could prepare larger vesicles than short-chain surfactants (Span 20 and Tween 20). The EE % of niosomes made of Span 60 was moderately greater than other evaluated Span series. This may be because Span 60 has the highest phase transition temperature (Tc, 53º C) and the longest saturated alkyl chain (C16). Proniosomes made with Spans permeated better than Tweens. The improved proniosomal formulation which contains “Span 60” exhibited enhanced flux with an increase of 5.3 times and presented comparable therapeutic efficacy in both anti-inflammatory and antinociceptive effects to oral marketed NPX tablets with the identical dose. The evidence presented here suggests that transdermal NPX therapy using proniosomes can be a viable alternative to oral therapy, avoiding undesirable side effects such as GI bleeding, perforation, and ulceration (Shah et al., 2021[252]).

#### Antimicrobials

##### Dapsone

Dapsone (4,40-diamino diphenyl sulfone) is an aniline derivative compound that belongs to the synthetic sulfone group. Dapsone is an antimicrobial agent originally used to treat leprosy, but it is now used to cure different dermatological conditions such as neutrophilic dermatoses, dermatitis herpetiformis, and vasculitis (Wozel and Blasum, 2014[300]). Dapsone represents both bacteriostatic and anti-inflammatory characteristics. It's antimicrobial effect relates to its sulfonamide-like capability to prevent the synthesis of dihydrofolic acid (Coleman, 1993[58]). In addition, dapsone shows numerous anti-inflammatory features. It directly prevents the creation of reactive oxygen species (ROS) and reversibly hinders the myeloperoxidase, thereby reducing the formation of hypochlorous acid (Webster et al., 1984[294]). Dapsone prevents the adherence of neutrophils that is mediated by beta-2 integrin (CD11b/CD18) and down-regulates interleukin 8 (IL-8), which has an important role in neutrophil-mediated inflammation. It also normalizes the functions of lymphocytes and monocytes (Booth et al., 1992[39]; Kanoh et al., 2011[124]). Dapsone inhibits prostaglandin synthesis and release in a dose-dependent way, in addition to interfering with the activity of other proteins in the integrin family (leukocyte-function-antigen, Mac-1, p150,95). Similarly, dapsone can inhibit TNF-α and generate leukotriene products in polymorphonuclear leukocytes (Wozel and Lehmann, 1995[301]). Regarding its impact on neutrophils, dapsone can reduce the effect of eosinophil peroxidase on mast cells, resulting in less histamine release. Finally, dapsone was revealed in *in vitro* studies to suppress the alternative pathway of complement activation, but this was not replicated *in vivo* (Hashimoto et al., 1984[105]; Bozeman et al., 1992[41]).

Acne vulgaris is a multifactorial inflammatory skin condition that usually inflicts adolescents and young adults and places a significant physical, psychological, and psychosocial problem on those who are affected (Zaenglein et al., 2016[308]). Acne is characterized by increased sebum production as a result of an androgenic impact on the pilosebaceous unit, which leads to a chronic inflammatory disorder, bacterial colonization by *Propionibacterium acne*, altered keratinization, and inflammation of hair follicles in numerous zones of the body such as the forehead, cheek, chest, neck, and back (Williams et al., 2012[296]). There is no typical acne treatment; however, an appropriate medical to improve lesions can be established for the majority of patients. When used in combination, suitable local applications of medications such as benzoyl peroxide, retinoids, and antibiotics typically manage to improve mild to moderate acne (Williams et al., 2012[296]). Dapsone, as an antibiotic, is normally combined with rifampin and clofazimine for leprosy treatment (Sago and Hall Iii, 2002[245]). Due to its low water solubility and AEs, such as hemolytic anemia, headache, and nausea, oral use of dapsone is restricted (Shamma et al., 2019[255]). Since the hematological AEs of oral dapsone is considerable, topical dapsone may be a preferred option in case of mild to moderate acne. Experimental studies of a topical gel formulation of dapsone (5 %) have shown its effectiveness in acne vulgaris handling (Stotland et al., 2009[271]). In 2005, the US Food and Drug Administration (FDA) accepted the first dapsone topical gel for acne (Aczone®, manufactured by Allergan, Inc.). This medicine is available in the United States in formulations containing 5 % and 7.5 % of the active ingredient for twice and once-daily use, respectively (Schneider-Rauber et al., 2020[248]). Shamma et al.*,* prepared dapsone gel using cyclodextrin (Shamma et al., 2019[255]). Also, other documents exhibited the topical dapsone formulation by different vesicular systems (El-Nabarawi et al., 2020[75]). 

Al Sabaa et al.*, *developed and evaluated the efficacy of dapsone niosomal formulation for topical application in order to control and extend the release of the drug with enhanced skin penetration. Niosomes were formed by utilizing the TFH process with varying ratios of surfactants (20, 40, 60, and 80), and cholesterol and niosomes sizes ranged from 2.25 to 6.32 μm (Hatem et al., 2018[107]). When they evaluated the mean scores of lesions (MSL) after two and eight weeks of treatment, a significant improvement was observed (P<0.001). Both inflammatory and non-inflammatory lesions have responded to the topical application of niosomal dapsone, significantly impacting the MSL (P< 0.001) (Hatem et al., 2018[107]).

In another study on the niosomal formulation of dapsone, Habib et al. (2022[102]) used a D-optimal mixture design to reach the best formulation of niosomes to use in *in vivo* acne models. The niosomes were made of Span 20, cholesterol, and cremophor RH. The optimized gel formula was developed by dispersing Carbopol^®^ 945 (1 %) in concentrated niosomal suspension. The average particle size of niosomes ranged from 95.6 to 320.7 nm, and the mean EE ranged from 72.5 to 86.3 %. *In vivo* experiments of the optimal gel formula for topical treatment of *Cutibacterium acnes* infected BALB/c mice revealed more results when compared to Aknemycin in both treated and untreated groups. In addition, significant inflammation reduction in percentage was observed from optimal niosomal gel formula compared to other groups. These findings demonstrated that the D-optimal mixture design was an effective means for optimizing the dapsone niosomal formula. Furthermore, this optimized gel formula effectively treated C. acnes-infected mice (Habib et al., 2022[102]). 

##### Erythromycin

Erythromycin is a macrolide antibiotic that hinders ribosomal protein biosynthesis by binding to the 50S ribosomal subunit. In recent years, it was shown that macrolides have anti-inflammatory effects beyond their bacteriostatic and bactericidal effects. Therapeutic concentrations of erythromycin reduce IL-8 mRNA expression in the patient's bronchial epithelial cells. Erythromycin also could influence immune cells such as neutrophils, lymphocytes, and monocytes.

NF-κB is known for regulating gene transcription, which tends to make inflammation mediators and related enzymes, such as iNOS, COX-2, TNF-α, IL-1, and IL-6. Studies represent both erythromycin and roxithromycin could inhibit NF-κB activation (Kwiatkowska and Maslinska, 2012[139]). Regularly applying topical erythromycin results in AEs such as skin redness, irritation, and itching. On the other hand, erythromycin niosomal formulation may enhance drug retention within the skin, increasing therapeutic effects and decreasing adverse effects (Jigar et al., 2011[120]).

In their study, Mohammadi et al.*,* compared the efficacy of niosomal erythromycin 4 % to erythromycin 4 % and zinc acetate 1.2 %. TFH technique was used to develop niosomal formulations, and the molar ratio of non-ionic surfactant (Span 60) and cholesterol was 7:3. All of the patients in this study showed a 75 % reduction in acne lesions belonged to the niosomal group (P = 0.002). However, despite a reduction in the number of acne lesions in both groups during the initial weeks of treatment, a slight increase in the number of lesions was detected at the end of the treatment, which could be attributed to the patients' poor adherence to the treatment and low compliance. Furthermore, the drug's efficacy in both treatment groups was higher in inflammatory lesions than in non-inflammatory lesions. This could be because topical antibiotics have a more substantial anti-inflammatory effect than comedolytic effect (Mohammadi et al., 2017[178]). 

##### Minocycline

Minocycline is a common antibiotic, a tetracycline derivative, and a broad-spectrum bacteriostatic agent. This antibiotic inhibits Gram-positive and negative bacteria and can successfully pass the blood-brain barrier. Tetracyclines connect to the bacterial 30 S ribosomal subunit and stop protein synthesis, which is the basis for their primary mechanism of antibacterial activity (Asadi et al., 2020[24]). Minocycline has a longer half-life and more excellent tissue absorption into the CNS and cerebrospinal fluid compared to first-generation tetracyclines. The greater effectiveness of minocycline is the result of the modification in ring D through carbons 7-9. FDA has primarily authorized this antibiotic to treat RA and several sexually transmitted illnesses (Blum et al., 2004[37]).

Minocycline has a better pharmacokinetic profile than first-generation tetracyclines due to its rapid and good tissue penetration, prolonged half-life, and high bioavailability when administered orally. The majority of recent reports have concentrated on minocycline's non-antibiotic characteristics (Barza et al., 1975[31]). Minocycline is a tetracycline with the best tissue penetration, which has a dimethylamino group at position seven instead of methyl and hydroxy groups at position five and is consequently more lipophilic than other tetracyclines (Colton et al., 2016[59]).

However, several types of research that were used in clinical settings reported on local minocycline delivery. A bacterial infection that results in inflammation and bone loss at and around the implant is the primary cause of most peri-implant disorders. In this work, niosomes of minocycline were produced by TFH method and applied to the dental implant as an antibacterial coating. Minocycline-loaded niosomes were evaluated for drug loading capacity, EE, and yield. The findings showed that the best formulation, consisting of cholesterol and Span 60 in a 1:3 ratio, had the highest drug-loading content and EE. In addition, drug release from the coated implants *in vitro* and antibacterial activities were investigated. According to the findings, the coated dental implant's ability to release minocycline could be managed for up to 7 days after implantation, which was a critical time frame. At this time, *Porphyromonas gingivalis* inhibition was also noted. According to an *in vitro* cytotoxicity test, the coated implant was shown to be non-toxic to osteoblasts. The results of this study represented the possibility of covering medical equipment with an antibacterial substance (Wongsuwan et al., 2020[299]).

#### Miscellaneous

##### Diacerein (DCN)

Diacerein (DCN) is a cleaned anthraquinone that is wholly converted during absorption into Rhein. DCN is part of a new category of anti-OA drugs known as "disease-modifying osteoarthritis drugs (DMOADs)" (Gadotti et al., 2012[90]). Its main mechanism of action is to suppress the IL-1 β system and relevant downstream signaling. Diacerein has been shown to influence IL-1 β activation via decreasing IL-1 β converting enzyme production, as well as to impact IL-1 β sensitivity by decreasing IL-1 β receptor levels on chondrocyte surfaces. In addition, it indirectly raises IL-1 β receptor antagonist production. DCN inhibits IL-1β-induced activation of NF-κβ, which stimulates pro-inflammatory cytokine expression (Pavelka et al., 2016[214]). DCN oral formulations have not been successful because of their unfortunate physicochemical properties (solubility 0.01 mg/mL) and an unacceptable pharmacokinetic profile, such as poor bioavailability (35 %-56 %) and short half-life (4 hours). It is recommended to be taken 40-50 mg twice daily orally. Patients typically become less compliant when they have to follow a twice-daily DCN regimen for the suggested treatment of 2-3 years. Furthermore, because of the high incidence of severe AEs related to long-term oral administration, the clinical application of DCN is limited (Rehman et al., 2015[237]). Because of all mentioned above, the topical route could be an appropriate choice to bypass AEs of DCN by using vesicular drug delivery systems like niosomes, and it is expected to enhance DCN absorption in the site of action efficiently and decrease its AEs.

El-Say et al. (2016[76]) examined the niosomal gel of DCN for topical application. The niosomes were made of non-ionic surfactants such as Tween 60, Tween 40, and a mixture of Tween 60 and Tween 40 with different percentages of stearyl amine as a charge-inducing agent. The size of DCN niosomes ranged from 7.33 µm to 23.72 µm and vesicles' EE ranged from 9.52 % to 58.43 %, which was improved by enhancing the percentage of stearyl amine from 0 to 10 %. Maximum released DCN from the formulation at the end of 8 hours (98.1 %) was related to a niosomal formulation which was made of Tween 60 with the lowest HLB and maximum encapsulated DCN. Niosomes were incorporated with different gel bases; for example, HPMC 4000, methylcellulose (MC), carboxymethyl cellulose (CMC), and Carbopol^®^ 934 NF (CRP). HPMC (3 %) and MC gel (3 %) formulations were the most suitable for the topical application of DCN. DCN niosomal gel's anti-inflammatory activity in rats was studied using carrageenan (CA)-induced hind paw edema. The positive control group treated with commercial gel (reference) showed maximum edema of about 20.83 % inhibition at 6 h. Both HPMC (3 %) and MC (3 %) DCN niosomal gels had higher anti-inflammatory activity than the reference gel (37.66 % and 34.52 % inhibition, respectively). The authors guess improvement could be credited to the niosomal system's smaller vesicle size, which would improve DCN permeability through the skin's multilayered structure. Furthermore, topical application of DCN niosomes in HPMC and MC gels to the inflamed hind paw of rats meaningly (p<0.05) reduced the edema size (El-Say et al., 2016[76]).

Moghadam et al.*,* examined the topical niosome of DCN used in psoriasis. Their results suggested that Span 60, cholesterol (9:1), and 45 minutes of hydration time were favorable for niosome preparation. EE of the optimal formulation was 83.02 % with a size of 477.8 nm. *In vitro* skin penetration study revealed that the maximum flux was related to the optimal formulation (2.820 μg/cm^2^/h) with 90 % of Span 60 and followed by the Higuchi model and non-Fickian transport mechanism, whereas the flux of formulation with 70 % of Span 60 was lower and was about 2.14 μg/cm^2^/h (Moghddam et al., 2016[175]; Aziz et al., 2018[26]). 

##### Ivermectin (IVM)

Ivermectin (IVM) is an antiparasitic drug. Nowadays, it is recommended to treat infestations, including head lice, scabies, river blindness (onchocerciasis), strongyloidiasis, trichuriasis, ascariasis, and lymphatic filariasis (Laing et al., 2017[140]).

Numerous studies related IVM effectiveness with early diagnosis and administration to tackle the intestinal phase, with restricted activity being noted against encysted larvae. A study was deployed to use niosomes for improving the efficacy of oral IVM against different stages of *Trichinella spiralis* infection with reference to nano-crystalline IVM. In addition to counting adult and larval worms, the jejunum and diaphragm were histopathologically examined to perform the assessment. Additionally, the biochemical status of oxidant/antioxidant, angiogenic, and inflammatory biomarkers in muscle and intestinal tissues was assessed. When compared to the infected, untreated control, both niosomes and nano-crystals significantly reduced the number of adults and larvae. This was due to the superior activity of niosomal IVM. Niosome activity was further demonstrated by a decline in inflammation in both jejunal and muscle homogenates. In comparison with infected, untreated control mice at various stages, biochemical parameters in all treated mice showed highly significant differences, with niosomal IVM having a particularly strong influence. In addition, in this study, the effectiveness of niosomal IVM in treating various phases of trichinellosis outperformed that of nano-crystalline IVM (Elmehy et al., 2021[73]).

##### Melatonin

Melatonin is a neurohormone with anti-inflammatory and antioxidant properties (Damrongrungruang et al., 2020[65]; Uthaiwat et al., 2021[288]). In a preliminary investigation conducted in 2021, glutaryl melatonin's potential application as a dermal anti-inflammatory medication was evaluated. The anti-inflammatory characteristics of *Candida albicans*, *Escherichia coli* lipopolysaccharide (LPS)-induced RAW cells, and a croton oil-induced ear edema model in ICR mice were explored. Intraperitoneal injections were used to cause mucositis in animals. Mice (10-week-old, n = 6/group) were given topical or oral treatments of melatonin (2 %w/w) or glutaryl melatonin (2 %w/w) niosomal gel. Then comparison took place between the mice of the control group, consisting of mice that received fluocinolone acetonide (0.5 %w/w) and blank conditions. Glutaryl melatonin has significant anti-inflammatory and anti-candidiasis activities. The integration of glutaryl melatonin in a niosomal gel formulation exhibited the ability of topical oral applications to decrease mouth discomfort caused by 5-FU treatment in mice (Damrongrungruang et al., 2020[65]).

The following study focuses on the efficacy of MNG (melatonin niosomes gel) in treating 5-FU-induced oral mucositis in mice. For this reason, 5-FU was used to produce oral mucositis in ICR mice, who were then randomly assigned to 4 groups. The first group received daily administrations of oral transmucosal MNG. The second group took a fluocinolone acetonide gel. The third group took a blank niosomal gel, and the last group remained with no treatment for five days compared to the control group. Other evidence on microscopic histopathology, proinflammatory cytokine levels, and oxidative stress markers were recorded. Treatment with MNG and fluocinolone acetonide did not result in very significant dissimilar histopathological, FTIR, interleukin-1β, or malondialdehyde (MDA) results in the tongues which were used as the oral tissue samples. As a result, topical MNG may reduce inflammation and oxidative stress in 5-FU-induced oral mucositis (Uthaiwat et al., 2021[288]). To optimize the pharmacokinetics of exogenous melatonin, further transmucosal niosomal gels were produced. MNG was described, and melatonin levels in healthy subjects were evaluated. Micron-sized MNG demonstrated identical *in vitro* release but variable *in vitro* permeability to melatonin gel, with a mean *ex vivo* residence period of more than 3 h and maximal adhesiveness at 25 and 37 °C. Oral transmucosal MN gels at 2.5, 5, and 10 mg were topically applied in 14 healthy individuals in a randomized, double-blind crossover design with a week washout. They demonstrated dose-proportional pharmacokinetics, with increased absorption and longer systemic circulation (Priprem et al., 2018[222]).

In conclusion, topical treatment of niosomal gel had an anti-inflammatory effect and enhanced oral wound contraction in rats, possibly due to enhanced mucosal permeability (Priprem et al., 2018[220]). 

##### Pentoxifylline (PTX)

Methyl xanthine is the source of the drug pentoxifylline (PTX). It is a well-known hemorheological agent as well as an effective TNF-α inhibitor. PTX can selectively reduce IL-2 and interferon (IFN) at optimal quantities, and at higher concentrations, it can overwhelm both cytokines produced by T Helper cells (Th1 and Th2). It also has vasodilator properties by increasing the amount of cyclic adenosine monophosphate (cAMP) in blood vessel smooth muscles (Schroer, 1985[249]; Basak and Ergin, 2001[32]). In 1984, FDA approved PTX as the first active pharmaceutical ingredient (API) for the suggestive management of intermittent claudication. According to reports, it reduces blood viscosity and fibrinogen, increases the deformability and flexibility of white blood cells, and slows platelet aggregation and neutrophil adherence (Angelkort et al., 1979[22]; Tjon and Riemann, 2001[281]; Murabito et al., 2002[187]; Alonso-Coello et al., 2012[18]). Because of its effect on blood rheology, it is utilized as an antithrombotic agent in ischemic illnesses. Other AEs of PTX include venous ulcers, burn scars, radiation-induced skin/soft tissue injury, and colorectal anastomosis wound healing activity. Because of its anti-inflammatory qualities and its inhibitory impacts on TNF-α and Th cells produced cytokines, PTX usage is advised in psoriasis (el-Mofty et al., 2011[74]; Hassan et al., 2014[106]; Ahmadi and Khalili, 2016[11]; Ghate et al., 2019[96]). According to a human investigation, PTX considerably reduces the Psoriasis Area Severity Index (PASI) compared to a placebo. After oral administration, PTX is entirely absorbed; however, due to first-pass metabolism, its bioavailability is only 20-30 %. The most fabulous alternative for treating skin disorders is topical application, but due to its hydrophilic nature, penetration through the skin is challenging. By selecting an appropriate colloidal drug delivery technology, such as niosome, for the problem, one can also improve patient compliance and reduce the needless exposure of the drug to other organs (Omulecki et al., 1996[196]; Teksin and Agabeyoglu, 2009[278]; Yewale et al., 2013[305]; Cavalcanti et al., 2016[48]; Alshehri et al., 2021[20]).

The majority of the drug administered in the PTX solution was left on the skin, according to the findings of a study on skin deposition. This is attributed to its hydrophilic nature, which prevents it from penetrating deeply into the skin. Niosomal formulations increased drug deposition in comparison to solutions. The considerable increase in drug deposition of PTX niosomes compared to the solution of PTX may be due to niosome adsorption in the skin and its effective skin diffusion. As a result, the PTX will be present in an adequate concentration at the site of action. The negative charge on the drug's surface may also have a role in the efficacy of PTX niosomal system penetration. In the dermis and lower hair follicles via stratum corneum (SC) and follicles, Ogiso et al.*,* noticed faster diffusion of negatively charged vesicles than positively charged vesicles (Ogiso et al., 2001[194]; Shahiwala and Misra, 2002[253]; Abdelbary and AbouGhaly, 2015[2]; Essaghraoui et al., 2019[80]; Bhardwaj et al., 2022[35]). 

The imiquimod (IMQ) model is one of the most popular mouse models for preclinical psoriasis research. On the skin, the model generates quick and repeatable responses. Bhardwaj et al.*,* investigated PTX-loaded niosomes. They prepared niosomes using the TFH technique. They used Swiss albino mice of either sex, weighing about 20-30 g, for an *in vivo* histological study to find out the efficacy of PTX niosomes. They divided the animals into four groups: Group 1 (normal mice), Group 2 (psoriasis-induced but untreated), Group 3 (psoriasis induced and treated with PTX solution), Group 4 (psoriasis induced and treated with PTX niosomes). The result showed that despite having excellent anti-inflammatory characteristics, PTX was unable to produce the desired response in group 3. It may be due to its hydrophilic nature, which prevents it from passing through the SC. The histology of group 4 showed improvement in terms of skin characteristics. It considerably reduced the thickened keratin layer, dermal hyperplasia, and skin irritation (Hawkes et al., 2017[108]; Bhardwaj et al., 2022[35]). This study used cholesterol, Tween 80, and soy lecithin to produce niosomes loaded with PTX for topical delivery in psoriasis. There was no PTX left on goat skin, which is used for the *ex vivo* permeation. PTX was much more accumulated in the SC and dermis in the case of niosomal formulation in skin deposition studies than what was observed in the drug's solution. In histological analyses, niosomal PTX was more efficient than PTX solution in reducing the generated inflammation. As a result, the produced niosomes loaded with PTX are suitable for transdermal drug delivery (Bhardwaj et al., 2022[35]).

## Natural Compounds

### Ammonium glycyrrhizate

*Glycyrrhiza glabra*, known as liquorice*,* is a valuable medicinal plant of the Fabaceae family (also known as Leguminosae). Although liquorice roots have been used since the Stone Age, it is now only found in Mediterranean countries such as Greece, Spain, and southern Italy. It has flavonoids (1-1.5 %) and triterpenoid saponins (3-5 %), primarily glycyrrhizin made up of calcium and potassium salts of 18-glycyrrhizic acid (also known as glycyrrhizic or glycyrrhizinic acid and a glycoside of glycyrrhetinic acid). The ammonium salt of glycyrrhizic acid (AG), in particular, has potent anti-inflammatory properties. Additionally, there is evidence that glycyrrhizin reduces inflammatory reactions brought on by spinal cord injury, including edema, tissue damage, apoptosis, and expression of inducible nitric oxide synthase (iNOS) and NF-κB, which facilitates the restoration of limb function (Table 3(Tab. 3); References in Table 3: Akbari et al., 2020[13]; Budhiraja and Dhingra, 2015[44]; Damrongrungruang et al., 2021[66]; Jamal et al., 2015[118]; Marianecci et al., 2012[163]; Marinho et al., 2021[164]; Meng et al., 2019[170]; Miatmoko et al., 2021[172]; Negi et al., 2017[192]; Pando et al., 2015[204]; Qiu et al., 2021[224]; Raafat and El-Zahaby, 2020[228]; Rezaeiroshan et al., 2020[238]; Sadeghi-Ghadi et al., 2021[243]; Song et al., 2014[268]) (Genovese et al., 2009[94]; Wang et al., 2015[293]; Rakhshandeh et al., 2023[231]).

The expression of TNF-α, IL-6, iNOS, and COX-2 was down-regulated in mice treated with glycyrrhizin, which significantly reduced the nociception brought on by acetic acid and formalin. Recent research suggests that glycyrrhizin's anti-inflammatory and antinociceptive effects depend on the substance's inhibition of the microglial high-mobility group box 1 protein (HMGB1) (Wang et al., 2015[293]; Sun et al., 2018[273]). Maione et al., carried out a major examination into the long-term analgesic and anti-inflammatory effects of ammonium glycyrrhizinate administration*.* According to this study, AG has anti-inflammatory and antinociceptive effects that last up to 48 hours after administration. Their findings implied that the inhibition of various pro-inflammatory cytokines and chemokines might be a contributing factor to its anti-inflammatory and antinociceptive effects. When considered collectively, all of these results show that AG is a long-acting therapeutic agent for the treatment of painful conditions and diseases associated with inflammation (Maione et al., 2019[153]).

According to the published literature, using novel drug delivery systems, such as non-ionic surfactant vesicles, can improve the compound's effectiveness as a potential anti-inflammatory drug and promote dermal pharmacological action. Marianecci et al. (2014[161])*,* reviewed the anti-inflammatory activity of novel ammonium glycyrrhizinate's niosomal delivery system. Glycyrrhizic acid and its derivatives as AG were recently confirmed to have anti-inflammatory properties in a mouse model with acute lung injury brought on by LPS. IL-10 and TNF-α levels were up-regulated by pretreatment with AG, while cAMP-phosphodiesterase (cAMP-PDE) activity was down-regulated in the lung tissue. In addition, PDE inhibitors are well known for their ability to stop the growth of edema when applied locally on a mouse paw.

In a clinical study, each volunteer's skin sites were pre-treated with a methyl nicotinate solution to cause erythema before applying formulations containing ammonium glycyrrhizinate at a concentration of 0.5 % (w/v). Different profiles of chemically induced erythema were developed depending on the anti-inflammatory formulation that was used. In comparison to the aqueous solution of ammonium glycyrrhizinate, the ammonium glycyrrhizinate-loaded niosomes were able to reduce the erythema much more quickly and effectively. The chemically induced erythema treated with ammonium glycyrrhizinate-loaded niosomes was actually reduced by 3.4-fold compared to the aqueous solution. The induced erythema vanished after 4 hours of treatment with ammonium glycyrrhizinate-associated niosomes. All skin areas that were exposed to the aqueous solution of ammonium glycyrrhizinate displayed intense erythema for up to five hours (Pieretti et al., 2006[217]; Shi et al., 2010[259]; Marianecci et al., 2012[163]).

In addition, to the mentioned study, Marianecci et al.*,* produced a niosome formulation of AG. The vesicles are composed of polysorbate 20 (Tween 20), cholesterol, and cholesterolesteryl hemisuccinate at various molar concentrations. In mouse models, the anti-inflammatory properties of AG-loaded NSVs were examined. NSVs were advantageous in comparison with the reference group in *in vitro* and *in vivo* studies. Additionally, based on their long-term stability, the anti-inflammatory AG medication unaffected the stability of the NSVs. When compared to AG alone and empty NSVs, *in vivo* tests showed that AG-loaded NSVs reduced edema and nociceptive responses more effectively. Results from *in vivo* and *in vitro* experiments showed that pH-sensitive and neutral NSVs are not significantly different (Table 3(Tab. 3)) (Marianecci et al., 2014[161]).

### Anthocyanins

Anthocyanins are natural substances that are extracted from a variety of food sources. They have recently been proved to have anti-inflammatory, antioxidant, and anti-candidiasis activities, suggesting that they may be effective for oral use (Vasconcelos et al., 2003[290]; Huang et al., 2014[112]; Sogo et al., 2015[266]; Petruk et al., 2017[216]). In previous research, two of the most effective anti-inflammatory anthocyanidins, cyanidin and delphinidin, created an anthocyanin complex (AC) that reduced TNF-α induced inflammation and inhibited candidiasis in healthy oral keratinocytes (Zhu et al., 2013[311]).

Damrongrungruang et al.*, *investigated AC complex niosomes and implemented a randomized block design, placebo-controlled double-blind clinical trial. The TFH method was implemented to develop AC niosomes. Most participants were females aged 18 to 25 or 46 to 60. The AC niosomal gel-treated group had the most prominent initial wound size on average, followed by the AC gel-treated, placebo-treated, and triamcinolone gel-treated groups, in that order (Zhu et al., 2013[311]; Damrongrungruang et al., 2021[66]).

This study found that average wound sizes in the AC niosomal gel-treated group decreased significantly on days 1 and 3 (P < 0.05). In contrast, average wound sizes in the AC gel-treated group were only reduced on day 3 (P < 0.05). There was no noteworthy difference between the placebo gel and triamcinolone gel groups. The pain score was meaningfully reduced in all treatment groups (P < 0.05). According to this study, erythema of the wounds was decreased in all treatment groups significantly. Quality of life scores for AC niosomal gel-treated individuals improved with each consecutive assessment and was better than for the placebo gel-treated group at the same time (P < 0.05) (Table 3(Tab. 3)) (Limsitthichaikoon et al., 2018[143]; Damrongrungruang et al., 2021[66]).

### Carnosine

Carnosine (β-alanyl-L-histidine) is a natural dipeptide. It is also available over-the-counter as a food extra. It has a well-established multimodal mechanism of action that contains the detoxification of ROS, the downregulation of the production of pro-inflammatory mediators, the prevention of the formation of abnormal proteins, and the modulation of cells in the peripheral (macrophages) and brain (microglia) immune systems (Baradaran Rahimi et al., 2019[30]; Caruso, 2022[47]; Farhadi et al., 2023[83]).

Many organisms naturally contain carnosine, which can be found in their hearts, brains, and muscles. The primary source is dietary intake (meat and fish), but carnosine synthase can also produce it internally (Boldyrev et al., 2013[38]). L-histidine and carnosine were found to enhance catalase expression, GSH content, and glutathione peroxidase (GPX) activity. They also stopped oxidative damage and inflammatory mediators caused by ethanol in a dose-dependent way (Liu et al., 2008[145]).

In the study of Tsai et al. (2010[285]), anti-inflammatory effects were investigated. The neuroprotective properties of carnosine were investigated in mice given treatments with 1-methyl-4-phenyl-1,2,3,6-tetrahydropyridine (MPTP). Carnosine was directly infused into the water supply for four weeks at concentrations of 0.5, 1, and 2 g/L. MPTP treatment significantly decreased the amount of glutathione in the striatum, decreased the activity of the antioxidant enzymes GPX, superoxide dismutase (SOD), and catalase, enlarged the levels of malondialdehyde and ROS, and increased the production of IL-6, nitrite, and TNF-α. Carnosine meaningfully reduced the amount of glutathione lost as a result of MPTP, preserved the activity of GPX and SOD, reduced oxidative stress, and decreased levels of inflammatory cytokines and nitrite while also suppressing iNOS activity (Tsai et al., 2010[285]).

Moulahoum et al.*,* explored the impacts of zinc supplementation on carnosine's antioxidant and anti-inflammatory activity. They showed that zinc supplementation could potentially boost carnosine's ability to protect proteins from structural and functional changes brought on by aging. Carnosine has been shown to produce a complex with zinc. Their findings demonstrated that zinc supplementation increased carnosine activity against protein glycation, oxidation, and aggregation in a dose-dependent manner (Moulahoum et al., 2020[185]).

Lu et al.*,* demonstrated the efficacy of the Fe_3_O_4_ nanoparticles/PLGA polymer as a drug delivery system for the simultaneous treatment of stroke and blood-brain barrier (BBB) crossing (Lu et al., 2021[148]).

The non-selective cation channel known as transient receptor potential akyrin type 1 (TRPA1) is essential for the perception of pain and neurogenic inflammation. The content of allipoic acid and carnosine combined in niosomes formulation was examined in the study by Maestrelli et al. (2019[151]). Their team has recently created a family of lipoic acid-based TRPA1 antagonists known as ADM-9 that can reverse both inflammatory trigeminal allodynia and neuropathic pain caused by oxaliplatin, which is particularly important in the treatment of orofacial pain and migraine pain. In their research, the activity of ADM on neocortex cultures was examined, and an effective formulation to cross the BBB was created to increase the concentration of ADM in the brain and quickly and selectively deliver it to the central nervous system (CNS) (Maestrelli et al., 2019[151]).

### Celastrol

Celastrol (CEL, also known as tripterine) is a natural friedelane pentacyclic triterpenoid isolated from Celastraceae plants such as *Tripterygium wilfordii* and *Celastrus orbiculatus*. *T. wilfordii* has been used in traditional Chinese medications to treat RA for centuries, and CEL has been known as one of the most important anti-inflammatory active ingredients. Additionally, widespread literature is available on CEL's remarkable anticancer potential (Xu et al., 2021[302]). CEL exerts its anti-inflammatory effects by regulating inflammation mediators and suppressing pro-inflammatory cytokines (IL-2, IL-6, IL-8, IL-1β, TNF-α, IFN-γ, etc.) and enzymes (NOS, iNOS, COX-1, COX-2, prostaglandin E2, etc.) via NF-κB prevention. CEL also inhibits inflammatory cell migration, proliferation, and activation. For instance, the nuclear receptor Nur77 may promote mitochondrial autophagy and inhibit the formation of neutrophil extracellular traps (NETs) and the neutrophil oxidative. Furthermore, other pathways, including the cannabinoid receptor-2 (CB2) signaling pathway and the RIP3/MLKL pathway, have been linked to CEL's anti-inflammatory properties (Hou et al., 2020[110]). CEL has promising anticancer and anti-inflammatory activities; however, the clinical application of CEL is limited because of its severe AEs. These AEs are primarily due to their unfavorable bio-distribution and numerous physicochemical and pharmacokinetic limitations, such as low water solubility and bioavailability; therefore, some strategies, such as vesicular systems, may be used to overcome these challenges (Shi et al., 2020[258]). Meng et al.*, *performed niosomal CEL in Carbopol^®^ 974-based gel and compared that with CEL gel in psoriasis-induced mice by commercially available imiquimod cream (IMQ, 5 %). The niosomes were composed of Span 60, Span 20, and cholesterol in a 3:1:1 weight ratio. The niosomes had a particle size of about 147 nm and a yield of up to 90 %. In comparison with the raw drug, niosomes enhanced CEL's *in vitro* permeation ability. CEL niosomes significantly reduced erythema on the dorsal skin of psoriasis mouse models in the *in vivo* study. Spleen weight and cytokine levels, including IL-17, IL-22, and IL-23, decreased following treatment, indicating that this formulation has a high therapeutic potential for psoriasis. Finally, formulating CEL in niosomal form increased its water solubility and skin permeation, effectively increasing its anti-psoriasis properties in mice (Meng et al., 2019[170]). Qiu et al.*, *conducted a similar study, and their research also revealed that following topical application of CEL Nio gel to mice, CEL was primarily accumulated in the skin rather than in the blood or lymphatic system, while inflammatory factors in the blood were significantly reduced. Furthermore, Nio preparation increased HaCaT cell uptake, whereas CEL clearly decreased inflammatory cytokine mRNA levels in HaCaT cells. The expression of Ki-67 is a marker for evaluating keratinocyte hyperproliferation in psoriasis treatment, so they used immunofluorescence to examine the expression of Ki-67 in IMQ-induced psoriatic mice. The result showed CEL Nio gel reduced the expression of the inflammatory cytokines and Ki-67 in the skin (Qiu et al., 2021[224]). 

### Curcumin (CUR)

Recent research suggests that curcumin (CUR) has anti-inflammatory and analgesic activities due to its significant prevention of pro-inflammatory mediators (Shah et al., 1999[251]; Park et al., 2012[210]; Ansari et al., 2023[23]). CUR has been shown in clinical trials to offer analgesic effects while maintaining a favorable safety profile (Eke-Okoro et al., 2018[69]). Oral administration of CUR (500 mg) three times per day revealed comparable efficiency to diclofenac (50 mg) administered twice daily, while CUR has a better safety profile among patients with knee OA. However, CUR's oral administration has been limited due to its poor aqueous solubility at acidic and physiological pH levels. Moreover, CUR is susceptible to degradation in an alkaline environment and low absorption and rapid metabolism, resulting in low bioavailability (Anand et al., 2007[21]; Khalil et al., 2013[129]; Naksuriya et al., 2014[188]; Shep et al., 2019[257]; Akbari et al., 2020[13]). Therefore, choosing a suitable drug delivery system for CUR is essential. Transdermal administration of CUR is one of the best and most practical methods. It has several advantages over oral drug delivery, including improved patient compliance in long-term treatment, avoiding first-pass metabolism, sustaining administration, maintaining a steady and prolonged drug level in plasma, limiting interpatient and interpatient variability, and allowing for treatment interruption or termination when necessary (Patel et al., 2009[212]). However, the chemicals' physicochemical characteristics and the vehicle's nature substantially influence the degree of penetration into the skin (e.g., the polarity of the solvent, particle size, and type of vehicle). CUR is an ideal medicine for transdermal delivery because of its molecular weight of 368.38 Da, melting point of 183 °C, and partition coefficient of (logP) 3.29 (Fernandez et al., 2000[88]; Chandrashekar and Shobha Rani, 2008[52]; Patel et al., 2009[212]).

CUR can influence gene expression related to cancer development, inflammation, cell survival, cell proliferation, invasion, and angiogenesis by blocking the activation of transcription factors. These elements include the proteins peroxisome proliferator-activated receptor-γ (PPAR-γ), activated protein-1 (AP-1), signal transducer and activator of transcription (STAT) proteins, NF-κB, and β-catenin. In addition, CUR's anti-inflammatory actions are achieved by inhibiting COX-1 and COX-2, which stops the eicosanoids prostaglandin E2 and 5-hydroxyeicosatetraenoic acid from being produced. In mouse models of colorectal cancer, suppression of these eicosanoids is similarly linked to a decrease in carcinogenesis (Huang et al., 1991[111]; Shishodia et al., 2007[261]; Fadus et al., 2017[81]).

Many scientists worked on CUR niosomes; for example, Alemi et al.*,* reported Paclitaxel and CUR niosomal formulations entered abnormal cells significantly more frequently than healthy cells (Alemi et al., 2018[16]). Katrolia et al.*,* investigated lycopene and CUR niosomes using ethanol injection (Table 3(Tab. 3)) (Katrolia et al., 2019[125]). The findings indicate that, in general, a rise in cholesterol concentration led to a rise in the rate at which CUR was released from niosomal formulations. The niosomal formulations with the highest cholesterol: surfactant ratios had rapid solubility, whereas those without cholesterol indicated the slowest dissolution. The addition of CUR in niosomal formulations can improve the dissolution of CUR, as evidenced by a comparison of the formulations with the control. This is consistent with permeation experiments, which indicated the best transdermal and dermal distribution for the niosomal formulation with the highest cholesterol: surfactant ratio. In comparison with CUR solutions, the result indicated that all curcusomal formulations had greater levels of penetration into and across the epidermal layers. It was predicted that the formulation of curcusome would not exhibit significant toxicity. More than 90 % of cancer cells were still alive after 24 hours of incubation with free CUR; however, only about 50 % of cells survived after treatment with 12.5 M of CUR-niosomes. When CUR was employed at a greater dosage (25 M), the cytotoxicity of CUR-niosomes was more effective (killing >80 % of cancer cells), whereas free CUR only killed 20 % of the cells (Akbari et al., 2020[13]).

It is common knowledge that CUR's ability to reduce pain has been regarded as a crucial pharmacological parameter. However, the main mechanism underlying CUR's antinociceptive effects on pain is not well understood. Therefore, medications like NSAIDs, which provide anti-inflammatory benefits by preventing COX-2 (inducible) and COX-1, can be used to reduce pain (constitutively active). Sadly, prolonged use of drugs that suppress COX-1 isozyme can lead to severe GI discomfort, ulcers, and kidney damage. Additionally, the protracted use of medicines that suppress COX-2 isozyme could result in significant cardiovascular-related morbidity and mortality. Contrary to conventional analgesics, CUR preferentially inhibited lipoxygenase (LOX), phospholipase A2, and COX-2, but not COX-1, resulting in the anti-inflammatory and analgesic profits without any AEs associated with conventional nonselective analgesics (Goel et al., 2001[97], Eke-Okoro et al., 2018[69]).

### Epigallocatechin gallate

Natural plants are a significant and reasonably priced source of useful medicines that can successfully treat various disorders. Green tea is made of tea plant leaves (*Camilla sinensis*), a vital source of many antioxidant polyphenols and frequently consumed as a traditional healthy beverage (Rashidinejad et al., 2014[234]; Dai et al., 2020[64]). Green tea leaves have several advantages. However, due to the oxidation of its compounds, black tea lacks these properties (Dai et al., 2020[64]).

Green tea's major polyphenolic compounds, which account for the majority of its antioxidant properties, are catechins. Research has linked epigallocatechin gallate to the numerous beneficial effects of green tea epigallocatechin gallate (EGCG) (Al-Sayed and Abdel-Daim, 2018[19]; Pandit et al., 2019[201]). The most significant green tea flavonoid, EGCG, has attracted the attention of innumerable investigators as a probable medication in the medical, dietary, and pharmaceutical fields. Numerous pharmacological properties of EGCG, including anti-tumor, antioxidant, and anti-inflammatory actions, have been discovered (Fujiki and Suganuma, 2012[89]; Grassi et al., 2013[99]; Gu et al., 2013[101]; Yu et al., 2014[306]). Additionally, it positively affects obesity, diabetes, Parkinson's disease, Parkinson's disease-related symptoms, and Alzheimer's disease (Crespy and Williamson, 2004[60]; Prasanth et al., 2019[219]). However, its bioavailability is relatively moderate (Baba et al., 2001[27], Cai et al., 2018[46]). This prevents it from being used as a medicine and causes a large discrepancy between the *ex vivo* and *in vivo* trials (Cai et al., 2018[46]). The poor bioavailability may be explained through its high hydrophilicity, which makes it difficult to penetrate cell membranes (Song et al., 2014[268]). Moreover, EGCG is unsteady and sensitive to environmental variables, including oxygen, pH variations, and other stressors (Cai et al., 2018[46]). Different formulation techniques for encapsulating EGCG are thought to effectively address these drawbacks (Dube et al., 2010[68]; Rashidinejad et al., 2014[234]; Song et al., 2014[268]), the anti-inflammation action of EGCG. Song et al. (2014[268]) investigated catechin and EGCG niosomes. Catechin and EGCG were tagged by fluorescein isothiocyanate (FITC) to evaluate the cell's drug absorption behavior. FITC-catechin and FITC-EGCG niosomes uptake values were 2.66 and 2.13 times greater than those not in niosomal form, respectively, at the maximal uptake level of 6 hours. The percentage of FITC-catechin, FITC-EGCG, and their niosomes that were taken up by Caco-2 cells was 0.67, 0.46, 1.72, 0.11, and 1.01 %, respectively (n=3). Some researchers, like Marianecci et al.*,* believe that nonionic surfactant, which is a part of the vesicle structure, acts as a penetration enhancer and may be responsible for the improved drug uptake (Marianecci et al., 2010[160]; Song et al., 2014[268]).

### Quercetin

Quercetin is a dietary flavonoid with many therapeutic benefits, including anti-inflammatory, antioxidant, anticancer, antibacterial, antiviral, and anti-diabetic characteristics (Smith et al., 2016[265]; Abdelkawy et al., 2017[6]; Sadeghi-Ghadi et al., 2020[244]). The ability of quercetin to reduce inflammation and oxidation is likely a result of its capacity to prevent the synthesis of critical inflammatory enzymes, including COX and LOX (Li et al., 2016[142]; Dahiya et al., 2019[63]). Furthermore, recent research has shown that hyaluronic acid conjugation can enhance the medicine's biological features, such as their stability and solubility (Mero and Campisi, 2014[171]; Abdel-Mohsen et al., 2019[7]). New genres of literature have also investigated hyaluronic acid's anti-inflammatory and antioxidant properties (Masuko et al., 2009[167]; Mendoza et al., 2009[169]). The use of hyaluronic acid in the present study's niosome structure for quercetin delivery was a novel method that improved drug loading and stability while improving the antioxidant and anti-inflammatory effects.

Sadeghi-Ghadi et al.*,* investigate quercetin with hyaluronic acid niosomes as a good formulation. They used the TFH method to prepare niosomes. The polymeric niosome's average size was 231.07 ± 8.39 nm (Sadeghi-Ghadi et al., 2021[243]). Quercetin-entrapped polymeric formulation's antioxidant activity was superior to that of empty polymeric niosomes. The antioxidant action of hyaluronic acid may be the cause of the approximately 10 % antioxidant impact of empty polymeric niosomes. Mohammed's study (2022[179]) exhibited that hyaluronic acid has a good antioxidant impact according to an *in vitro* test (Mendoza et al., 2009[169]; Sadeghi-Ghadi et al., 2019[242], 2021[243]; Witika et al., 2021[297]; Mohammed and Niamah, 2022[179]). According to the research, polymeric formulations exceeded free quercetin and empty polymeric niosomal dispersion regarding anti-inflammatory efficiency. Empty vesicles had an anti-inflammatory impact of about 10 % (Sadeghi-Ghadi et al., 2021[243]). The tissue anatomy of the layers under the epidermis is normal in the control group. A noteworthy number of elongated fibroblast cells were found in the connective tissue. The group receiving quercetin-entrapped polymeric niosomes had a similar tissue structure to the control group. Carrageenan (CA) causes severe acute inflammation by diffusion through the polymorphonuclear and mononuclear inflammatory cells. In the tissue structure of the layers beneath the epidermis, all groups received different dispersion/suspension formulations. In the CA group, the inflammation was more severe. In the group that received a basic suspension of quercetin, there was significant inflammation as well as some deposits. In quercetin-entrapped polymeric niosomes, inflammatory cell infiltration is reduced. CA injection resulted in large inflammatory cell infiltration when compared to empty polymeric niosomes as a blank and simple quercetin solution. The anti-inflammatory strength of quercetin-entrapped polymeric formulations outperforms quercetin-simple solutions and empty polymeric niosomes. To maintain the efficacy of polymeric niosomes, quercetin-entrapped polymeric niosomes suppress inflammation for a longer period than quercetin in suspension alone. Additionally, the approximately 10 % anti-inflammatory strength of empty polymeric niosomes may be a result of the anti-inflammatory strength of hyaluronic acid, which has been described. The produced vesicles showed considerable improvement; therefore, this formulation could be taken into consideration for more trials (Masuko et al., 2009[167]; Brusini et al., 2020[43]; Sadeghi-Ghadi et al., 2021[243]).

### Resveratrol (RSV)

Resveratrol (RSV) is a natural polyphenol found in many plants and has anti-oxidant, anti-inflammatory, cardioprotective, and anti-tumor characteristics. It has both chemopreventive and therapeutic activities. However, its applications are limited due to its quick oxidation, low water solubility, limited biological half-life, and quick metabolism and elimination (Caddeo et al., 2013[45]; Pando et al., 2013[202][203]; Scognamiglio et al., 2013[250]; Matos et al., 2014[168]). Additionally, due to its high photosensitivity, the molecule changes irreversibly from its active trans-isomer to its inert cis-isomer when exposed to light. Therefore, trans-resveratrol needs to be encapsulated before being applied topically or in food. Nevertheless, RSV is thought to be an intriguing medication to be used in dermatological preparations due to its chemoprotective and antioxidant capabilities. Its topical application in many physiological and pathological situations, such as preventing skin cancer or treating psoriasis, has received special attention (Jang et al., 1997[119]; Caddeo et al., 2013[45]; Scognamiglio et al., 2013[250]). The high drug concentration is available at specific action locations thanks to the transdermal administration. This is the reason that numerous studies on the *ex vivo* transdermal delivery of RSV in various nanocarriers have been conducted in recent years(Sinico and Fadda, 2009;[264] Pando et al., 2013[202]; Scognamiglio et al., 2013[250]; Marianecci et al., 2014[159]).

Many scientists investigated resveratrol niosomes. Pando et al. (2015[204]) used TFH-sonication and EIM to prepare niosomes. The effect of various formulation variables and preparation techniques on the release of RSV entrapped in niosomes across several epidermal barriers and anatomical structures was investigated. Diffusion of the active substance through the skin layers into the receptor phase, i.e., systemic fluids and blood vessels-is the primary mechanism of skin penetration (Pando et al., 2013[202]). As pig skin is recognized as a good alternative for *ex vivo* permeation studies due to its similarities with the SC of human skin regarding lipid structure, release research was conducted using the skin of newborn pigs. Even though there is a noticeable difference in thickness, young pig SC is much thinner than mature pigs and more resemblant to human skin while having more hair follicles (Pando et al., 2013[202]). Newborn pig skin has been the subject of numerous investigations, demonstrating its suitability for skin permeation tests (Marianecci et al., 2014[159]). When RSV reaches the inner tissues, which requires crossing the SC, RSV's effect on the skin becomes apparent. Three components were identified in this study: SC, epidermis, dermis (EDD), and receptor fluid (RC). RP-HPLC determined how much RSV had accumulated in these components (SC, EDD, and RC). The quantity of fatty acid used and RSV penetration was shown to be closely related (p < 0.05), with the relationship being stronger for niosomes made using the EIM and for both penetration enhancers, OA and LA. Thus, when this technique was used, there was reduced RSV buildup in the SC. Additionally, there was a clear relationship (p 0.05) between RSV permeation in EDD and the niosome preparation method, with the EIM being more successful among all formulations evaluated. The most successful niosomes were made using this procedure, with RSV penetration values up to 21 % and a weight ratio of 1:1 for both permeation enhancers. The RSV development in the SC in these individuals ranged from 5.4 to 27.7 %.

Negi et al.*,* prepared RSV niosomes using TFH and EIM. In the saline control group (Group 1), paw volume increased quickly and steadily (i.e., edema), and the inflammation persisted for the whole 12-hour investigation. The percentage of increase in paw volume was less in the groups receiving effective formulations (Groups 2, 3). Compared to the control group, it was revealed that the resveratrol formulation had an anti-inflammatory effect. Compared to ordinary gel, resveratrol-loaded niosomal gel was found to suppress edema consistently by a greater percentage (DS). The inhibitory profile for both the produced niosomal gel and the traditional gel formulation was essentially the same. In comparison with the commercial anti-inflammatory gel formulation, the resveratrol-entrapped niosomal gel effectively reduced the edema and demonstrated extended therapeutic efficacy. This can be attributed to their improved interface with skin constituents and capacity to establish skin depots. According to our research, niosomal hydrogel could be used as a topical formulation, which might increase the use of resveratrol as an anti-inflammatory agent (Negi et al., 2017[192]).

### Rosmarinic acid (ROSA)

Rosmarinic acid (ROSA) is a phenolic compound that is found in herbs and has beneficial effects such as anti-viral, anti-bacterial, anti-oxidative, anti-apoptotic, anti-tumorigenic, and anti-inflammatory effects (Luo et al., 2020[149]; Roohbakhsh et al., 2020[240]; Farhadi et al., 2023[83]). 

In 2015, Budhiraja performed *in vivo* research on the anti-inflammatory effect of ROSA niosomes (Budhiraja and Dhingra, 2015[44]). Niosomes of ROSA were developed by the REV method using different ratios of Span 85 and cholesterol. The research was conducted on skin inflammations like acne, performed on albino mice. To verify the safety of topical formulations, a skin irritation test was performed for both drug-loaded niosome and ROSA solution. It was found that the niosomal gel was less irritant than the ROSA solution. Parameters such as inflammation and bacterial multiplication rate were examined to estimate the efficiency of the formulations. In addition, they measured the skin's thickness to determine if there was a noteworthy difference between the drug-loaded niosomal gel group and the RA solution. RA-loaded niosomal gel suppresses inflammation more than ROSA solution. In 3 days, niosomal gel suppressed the inflammation by 58.16 %; however, ROSA solution inhibited inflammation by 40.9 % in 3 days. Also, only niosomal gel significantly affects colony count. The results showed that drug-loaded niosomes have anti-inflammatory effects, and niosomes are the appropriate carrier for the delivery of anti-microbial agents.

Another research in 2021 was performed to investigate the anti-inflammatory effect of niosomes loaded with ROSA on inflammatory bowel disease syndrome (IBD) (Marinho et al., 2021[164]). Niosomes loaded with RA recovered body weight loss and improved inflammation by increasing mucus production, decreasing myeloperoxidase activity, and decreasing expression of inflammasome components such as caspase-1, adaptor protein (ASC), and NLR family pyrin domain-containing 3 (NLRP3). Also, treatment with drug-loaded niosomes increased nuclear factor erythroid 2-related factor 2 (Nrf2) antioxidant signaling pathway and decreased TNF-α expression, although these effects were not identified with the RA-free treatment. The histopathological study of the colon of mice showed a high inflammation score in the DSS group with mucosal damage and an immense inflammatory infiltrate. Free RA affects colitis low and slightly recovers colitis and mucous secretion. Also, free RA and RA-loaded niosomes reduced colonic MPO activity, a marker for inflammatory cell infiltration. The marker for identifying the colonic damage was TNF-α which was increased in the DSS group and decreased in free RA and drug-loaded niosomes' groups. Additionally, the RA-loaded nanovesicles and DSS groups significantly differed in inflammasome-related protein amounts such as caspase-1, ASC, and NLRP3. The findings showed that RA-loaded niosomes have anti-inflammatory effects in mice-induced acute colitis.

### Stylopine and sanguinarine

A little plant known as “smoke of the earth” or *Fumaria* *officinalis* (FO), belonging to the *Papaveraceae* (*Fumariaceae*) family, is found in numerous Eastern Mediterranean regions. It has been utilized in Asian folk medication for a variety of painful and inflammatory conditions, including RA and conjunctivitis (Rakotondramasy-Rabesiaka et al., 2007[232]; Ivanov et al., 2014[116]; Khamtache-Abderrahim et al., 2016[130]; Fatima et al., 2019[85]). Furthermore, research has demonstrated its efficacy as an antioxidant, antiviral, and antibacterial agent (Orhan et al., 2004[197]). Furthermore, the plant's phytochemistry contains numerous secondary metabolites, particularly quinoline alkaloids (Manske, 1950[158]; Šimánek et al., 1999[263]; Sturm et al., 2006[272]). Therefore, it was interesting to investigate FO effects on different inflammatory and metabolic disorders and associated complications because of the plant's traditional usage in treating various chronic illnesses (Raafat and El-Zahaby, 2020[228]).

Raafat et al.*,* investigated *Fumaria* niosomes. The EIM was used to generate a variety of niosomal compositions. The anti-inflammatory capacities of FO, Alkaloid rich fraction (ARF), Stylopine (Sty), Sanguinarine (San), and other formulations were assessed in two different ways: acutely using the CA-induced inflammatory-pain method and chronically using the hind-paw edema method. In both cases, Ibuprofen 100 mg/Kg was used as a positive control (Boukhary et al., 2016[40]; Raafat and El-Zahaby, 2020[228]). The study indicated that the highest dosages of FO (200 mg/Kg), ARF (60 mg/Kg), Sty (30 mg/Kg), and San (30 mg/Kg) improved acute paw withdrawal thresholds in non-diabetic mice by approximately 17.20, 24.00, 119.99, and 115.89 times, respectively. These findings suggested that FO and ARF considerably reduced acute CA-induced inflammatory pain, as had been shown in the past with comparable substances (Raafat, 2019[227]). Additionally, acute paw withdrawal thresholds have improved by around 22.80 (Nio-1), 27.98 (Nio- 2), 25.87 (Nio-3), 19.60 (Nio-4), 23.99 (Nio-5), and 24.80 (Nio-6) fold, respectively, using niosome formulations. In acute CA-induced inflammatory pain studies, placebos I (drug-free Nio-3) and II (drug-free Nio-6) did not exhibit any noteworthy modifications. Using a previously described modified approach, the chronic hind-paw edema procedure was carried out (Boukhary et al., 2016[40]). It also kept an eye on how the potential of the investigated substances affected long-term anti-inflammatory reactions. The highest doses of FO (200 mg/Kg), ARF (60 mg/Kg), Sty (30 mg/Kg), and San (30 mg/Kg) reduced hind-paw edema by 82.72, 82.90, 72.78, and 64.54 %, respectively, using MRPP plethysmograph. According to these data, FO and ARF greatly reduced the chronic hind-paw edema brought on by CA. This has been shown with related natural phytochemicals (Boukhary et al., 2016[40]; Raafat et al., 2019[226]; Raafat, 2019[227]). Additionally, niosome formulations reduced a 59.09 (Nio-1), 89.99 (Nio-2), 85.45 (Nio-3), 57.27 (Nio-4), 78.18 (Nio-5), and 75.46 (Nio-6) percent in hind-paw edema, respectively. The chronic hind-paw edema studies with placebo I, and II did not reveal any noteworthy effects. This research showed that the long-term anti-inflammatory drug Nio-2 was the most effective at reducing the symptoms of chronic inflammation (Raafat and El-Zahaby, 2020[228]). The level of inflammatory mediators was assessed, as was done previously with the same natural products, to comprehend the anti-inflammatory mechanism of action credited to FO and its active phytochemicals (Choi et al., 2012[57]).

According to the anti-inflammatory data, Nio-2 had anti-inflammatory properties, which were most effective at reducing acute inflammatory pain (Rozza et al., 2014[241]; Raafat, 2019[227]). The present research supports the widespread use of FO as a traditional herbal remedy for acute and chronic pain, inflammation, and neuropathy. The ARF, which contains the two primary alkaloids Sty (48.1 %) and San (51.6 %), was found to be the most active fraction by *in vivo* bio-guided fractionation and chromatographic phytochemical analysis. The most optimized niosomal formulation was Nio-2 (Span 60, cholesterol: surfactant ratio of 1:2), according to *in vitro* optimization, analytical, and *in vivo* biological studies. By generating acceptable EE, quick degradation, and tolerable stability in simulated GI circumstances, this improved niosome, Nio-2, improved the pharmacokinetic characteristics of ARF. In this study, the most effective anti-diabetic and anti-inflammatory substances were FO, ARF, and Nio-2. Additionally, the major mechanism behind their antinociceptive and anti-inflammatory actions may involve a decrease in pro-inflammatory TNF-α and IL-6, a rise in levels of the anti-inflammatory cytokine IL-10, and a reduction in oxidative stress. When comparing similar concentrations, Nio-2 has demonstrated more efficacy than ARF. For additional investigation, this study may provide a potential oral formulation that effectively treats a variety of inflammatory disorders and diabetic consequences, particularly neuropathic pain (Raafat and El-Zahaby, 2020[228]).

### Trans Ferulic Acid (TFA)

Recent studies demonstrated that Trans Ferulic Acid (TFA), an aromatic compound that is abundant in fruits and foods, possesses biological properties such as anti-cancer, antioxidative, antimicrobial, and anti-inflammatory effects (Rezaeiroshan et al., 2020[238]). Yet, its effectiveness is restricted because of its low water solubility. Previous studies tried to overcome this issue with ferulic acid nanoparticles, TFA solid lipid nanoparticles, and ferulic acid nano-emulsions (Trombino et al., 2013[284]; Harwansh et al., 2015[104]; Panwar et al., 2016[205]). For enhancing its effectiveness, niosomes may be the key to solving this problem. 

Rezaeiroshan et al.*,* studied the anti-inflammatory impact of TFA-loaded niosomes on rat paw edema induced by CA. To form drug-loaded niosomes, they used the TFH method. They reported that the niosomal gel of TFA is significantly more effective in suppressing edema than conventional gel. Niosomal gel of TFA inhibits 21.37 % of edema, while conventional gel inhibits approximately 15 %. In addition, they demonstrated that the niosome is a proper carrier for TFA (Rezaeiroshan et al., 2020[238]).

### Ursolic acid (UA)

Ursolic acid (UA) is a pentacyclic triterpenoid obtained from plants such as apples, bilberries, basil, cranberries, rosemary, peppermint, and oregano (Farhadi et al., 2023[83]). In a variety of experiments, UA has been shown to have antioxidant, anti-inflammatory, anti-proliferative, anti-cancer, anti-mutagenic, anti-hypertensive, anti-atherosclerotic, anti-leukemic, and antiviral activities. It was found to prevent the activity of inducible nitric iNOS and COX-2 in RAW264.7 cells (mouse monocyte macrophage cell line). The anti-proliferative, anti-tumor, and anti-leukemic activities have been demonstrated to be facilitated by reducing the expression of NF-κB controlled genes such as LOX, COX-2, MMP-9, and iNOS (Checker et al., 2012[54]; Chan et al., 2019[51]). UA is water-insoluble and bioavailable. Based on the biopharmaceutical categorization system, UA is a class IV medication due to its low solubility in water and difficulties permeating biological membranes. These medicines have delayed disintegration and limited GI mucosa penetration, resulting in poor oral bioavailability. Drug delivery systems have been created to enhance the biopharmaceutical characteristics of this UA molecule. Mesoporous silica nanoparticles, nano-emulsions, solid lipid liposomes, nanoparticles, niosomal gels, and solid dispersions have effectively transported UA (Jinhua, 2019[121]).

Jamal et al.*, *investigated the niosomal gel system of UA based on proniosome technology for transdermal delivery. The optimized ursolic acid niosomal formulation (UANF) was dispersed in Carbopol 934 (1 %), and both analgesic and anti-arthritic activity were examined. The formulation, which was composed of Span 60 (85 mg), cholesterol (12.3 mg), and phospholipid (65 mg), met the requirements for a UANF. It was shown that UANF had a vesicle size of 665.45 nm, EE of 92.74 %, and a transflux rate of 17.25 g/cm^2^/h. Based on the *in vivo* bioactivity, UANF-gel could offer a respectable level of anti-arthritic activity as UA permeates through the skin more efficiently than standard gel (Omni gel). Furthermore, UANF-gel was found to be operative in treating arthritis based on a radiographic image. In order to treat arthritis and musculoskeletal disorders, niosomal gels of ursolic acid could be an operative and safe substitute for conventional therapy (Jamal et al., 2015[118]).

In another study, Miatmoko et al.*, *prepared UA niosomes which were coated by chitosan. According to the findings, increasing the quantity of UA was associated with an increase in the particle size of UA niosomes. However, the EE decreased when the quantity of UA added to niosomes increased. The maximum physical stability was attained by creating niosomes with a zeta-potential (ZP) value of -41.99 mV using a molar ratio of 3:2:10 for Span 60, cholesterol, and UA, respectively. Adding chitosan increased particle size from 255 nm to 439 nm, and ZP changed from -46 mV to -21 mV. In addition, UA niosomes coated by chitosan (Nio-UA-CS) exhibited more drug release in PBS at pH 6.8 and 7.4 than plain UA niosomes. The distribution of UA niosomes in the plasma and organs of the individuals was shown by the concentration and strength of coumarin-6. Nio-UA-CS gathered to high levels in the liver. Using coumarin-6 labeling, the chitosan coating on UA niosomes boosted their *in vivo* biodistribution in plasma and mouse tissues, according to the results of this study. However, this study has not verified the relation between both coumarin-6 and UA concentration as an active element in treatment (Miatmoko et al., 2021[172]).

## Conclusion

In recent decades, nano and micro-sized vesicular drug delivery systems have attracted researchers' attention. Most studies concluded that vesicular drug delivery systems could increase the stability of drugs that are sensitive to environmental conditions or particularly could protect them in harsh conditions of the GI tract for oral drug delivery. Furthermore, vesicular systems could increase the absorption of drug molecules with low absorption properties. Niosomes, a type of vesicular delivery system made of a non-ionic surfactant, were first developed for cosmetic and industrial use. They showed good stability compared to lipid bilayers and didn't need special conditions for manufacturing as needed in producing liposomes (for example, vacuum condition or nitrogen gas). Therefore, these vesicular systems are attractive to many researchers. However, in the case of anti-inflammatory agents, which include chemical and natural compounds, they mostly have absorption challenges and also have serious AEs. In the future, these formulations will have many applications in the field of targeted drug delivery, especially in the area of cancer, due to their physicochemical properties and biological effects. Polymers can be placed on the surface of niosomes to prepare targeted carriers. Their use, however, has limitations, such as high production costs. In conclusion, this literature indicates that niosomes could increase anti-inflammatory effects and plasma concentration of these compounds in animal models in different routes of administration, such as oral and dermal or transdermal delivery.

## Notes

Mohammad Saleh Fadaei, Mohammad Reza Fadaei and Pouria Rahmanian-Devin contributed equally as first author.

Ali Nokhodchi and Vahid Reza Askari (Applied Biomedical Research Center, Mashhad University of Medical Sciences, Azadi Sq., Vakil Abad Highway, Mashhad, 9177948564, Iran; Phone: +98 5138848931, Fax: +98 5138829279, E-mail: askariv@mums.ac.ir, vahidrezaaskary@yahoo.com, vahidrezaaskary@gmail.com) contributed equally as corresponding author.

## Declaration

### Competing interests

The authors declare no conflict of interest.

### Funding

The article received no funding.

### Authors' contributions

MSF, MRF, AEK, PRD, MMD, KNT, and AS wrote this manuscript and prepared the figures and tables. MSF, HH and VBR modified this manuscript and adjust the format. AN and VRA designed and supervised this project. All authors contributed to the article and approved the submitted version. All authors have read and agreed to the published version of the manuscript. 

## Figures and Tables

**Table 1:**
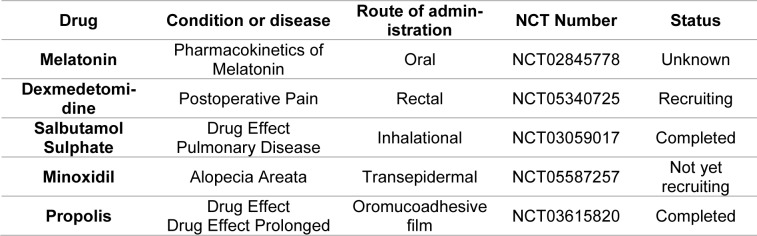
Clinical trials investigated on niosomal formulations

**Table 2:**
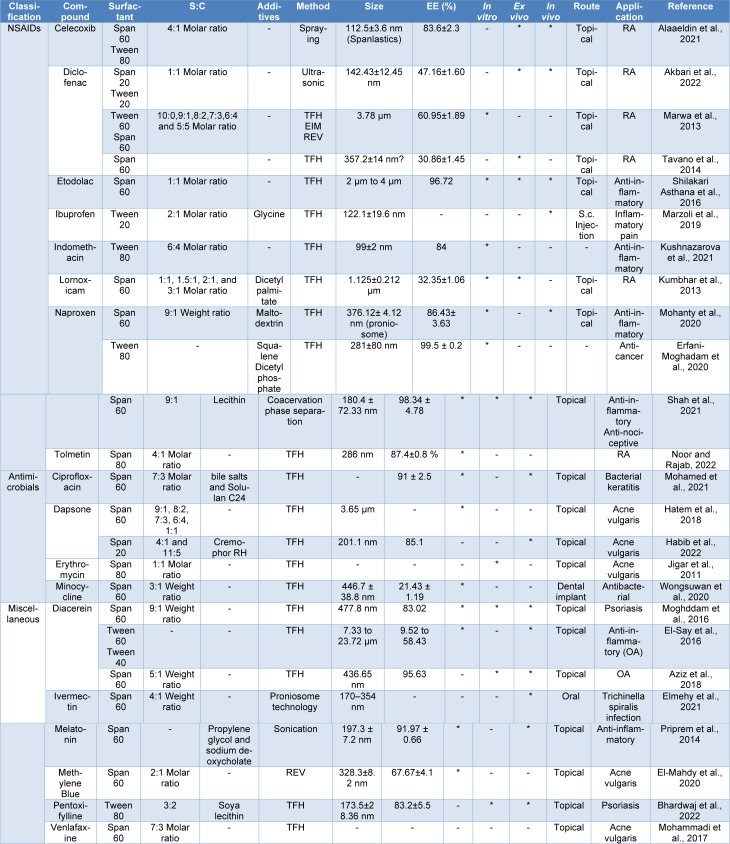
Niosomes of chemical compounds with anti-inflammatory effects

**Table 3:**
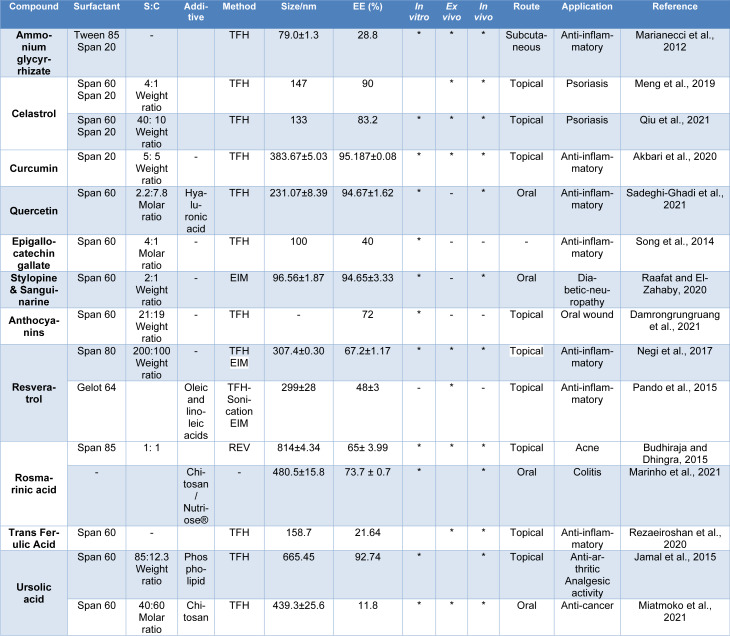
Niosomes of natural compounds with anti-inflammatory effects

**Figure 1:**
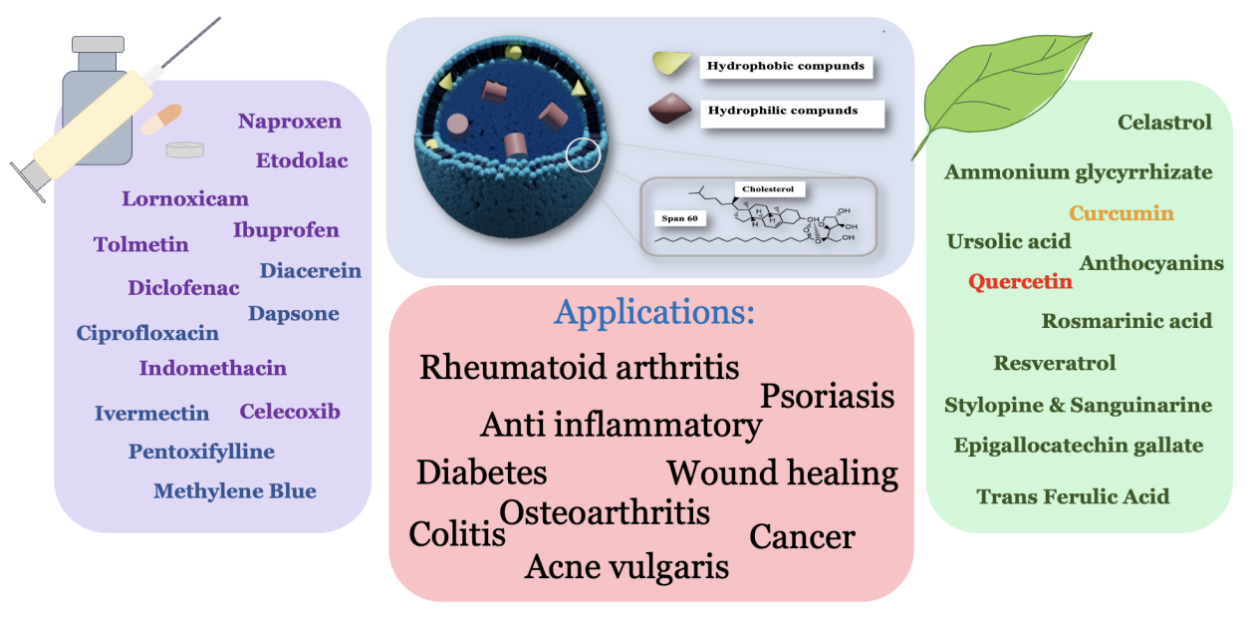
Graphical abstract
